# Multiple activities of sphingomyelin synthase 2 generate saturated fatty acid– and/or monounsaturated fatty acid–containing diacylglycerol

**DOI:** 10.1016/j.jbc.2024.107960

**Published:** 2024-11-05

**Authors:** Chiaki Murakami, Kamila Dilimulati, Kyoko Atsuta-Tsunoda, Takuma Kawai, Sho Inomata, Yasuhisa Hijikata, Hiromichi Sakai, Fumio Sakane

**Affiliations:** 1 Department of Chemistry, Graduate School of Science, Chiba University, Chiba, Japan; 2 Institute for Advanced Academic Research, Chiba University, Chiba, Japan; 3 Department of Biosignaling and Radioisotope Experiment, Interdisciplinary Center for Science Research, Organization for Research and Academic Information, Shimane University, Izumo, Japan

**Keywords:** diacylglycerol, sphingomyelin synthase, phospholipase C, phosphatidylcholine-phospholipase C, phosphatidylethanolamine-phospholipase C, ceramide phosphoethanolamine synthase, ceramide, D609

## Abstract

Phosphatidylcholine (PC)-specific phospholipase C (PC-PLC) (EC 3.1.4.3) and phosphatidylethanolamine (PE)-specific PLC (PE-PLC) (EC 3.1.4.62), which generate diacylglycerol (DG) and are tricyclodecan-9-yl-xanthogenate (D609)-sensitive, were detected in detergent-insoluble fractions of mammalian tissues approximately 70 and 35 years ago, respectively. However, the genes and proteins involved in PC-PLC and PE-PLC activities remain unknown. In a recent study, we observed that mammalian sphingomyelin synthase (SMS) 1 and SMS-related protein display PC-PLC and PE-PLC activities *in vitro*. In the present study, we showed that human SMS2, which is located in detergent-insoluble fractions of the plasma membrane, also possesses PC-PLC activity (approximately 41% of SMS activity), PE-PLC activity (approximately 4%), ceramide phosphoethanolamine synthase (CPES) activity (approximately 46%), and SMS activity in the presence of phospholipid-detergent mixed micelles. Moreover, purified SMS2 reconstituted in detergent-free proteoliposomes (near-native environments) showed PC-PLC, PE-PLC, and CPES activities. Notably, in the presence of approximately 2 mol% ceramide and 4 mol% PC (1:2 ratio), PC-PLC activity was almost equal to SMS activity. SMS2 as PC/PE-PLC showed substrate selectivity for saturated fatty acid– and/or monounsaturated fatty acid–containing PC and PE species. The PC-PLC/SMS inhibitor D609 inhibited all enzyme activities (SMS, PC-PLC, PE-PLC, and CPES) of SMS2. Moreover, Zn^2+^ strongly inhibited all the enzymatic activities of SMS2. Interestingly, diacylglycerol inhibited the SMS activity of SMS2 (feedback control). These results indicate that mammalian SMS2 has unique enzymatic properties and is a candidate for a long-sought mammalian PC/PE-PLC.

Diacylglycerol (DG) is a well-known lipid second messenger that activates PKC (1, 2). Moreover, DG regulates a wide variety of signal transduction proteins, including protein kinase D, β2-chimaerin, Unc-13, and Ras guanyl nucleotide-releasing protein (3, 4, 5, 6). DG is generated from glycerolipids, such as monoacylglycerol and triacylglycerol (7, 8, 9), through the action of monoacylglycerol-O-acyltransferases and lipase (adipose triglyceride lipase) (10) and triacylglycerol hydrolase (11)), respectively. In addition, DG is produced from glycerophospholipids, such as phosphatidic acid (PA), phosphatidylinositol (PI) 4,5-bisphosphate (PI(4,5)P_2_), phosphatidylcholine (PC), and phosphatidylethanolamine (PE), through the catalysis of PA phosphatase (PAP, lipin)/lipid phosphate phosphatase (LPP, PAP2) and phospholipase Cs (PLCs) (12, 13, 14, 15, 16). Furthermore, DG can be produced from PC and PE through sphingolipid metabolism including sphingomyelin (SM) synthesis and ceramide phosphoethanolamine (CPE) synthesis, respectively (17) (Figs. 1 and 2).

**Figure 1:**
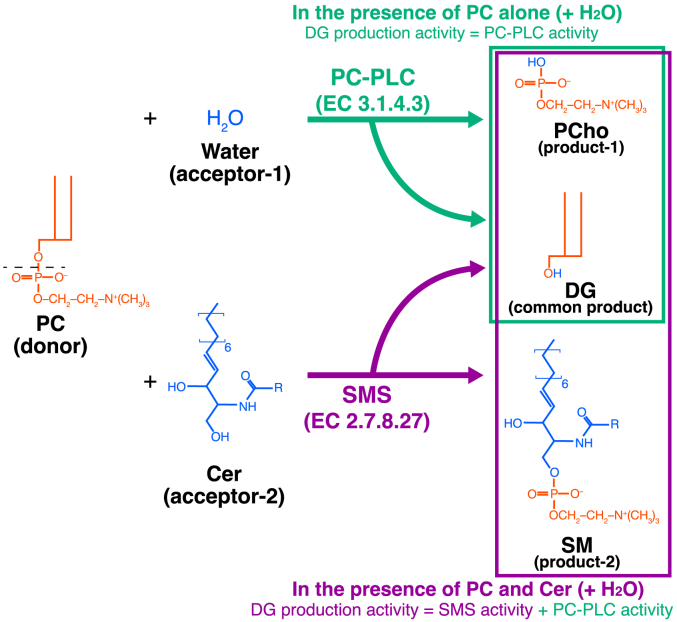
**Reactions of PC-PLC and SMS.** PC-PLC (EC 3.1.4.3) utilizes PC and water molecules to produce DG and PCho. SMS (EC 2.7.8.27) utilizes PC and Cer to produce DG and SM. In the presence of PC, Cer, and water, SMS2 can exhibit both PC-PLC and SMS activities. Cer, ceramide; DG, diacylglycerol; PC, phosphatidylcholine; PCho, phosphocholine; PLC, phospholipase C; SM, sphingomyelin; SMS, sphingomyelin synthase.

**Figure 2:**
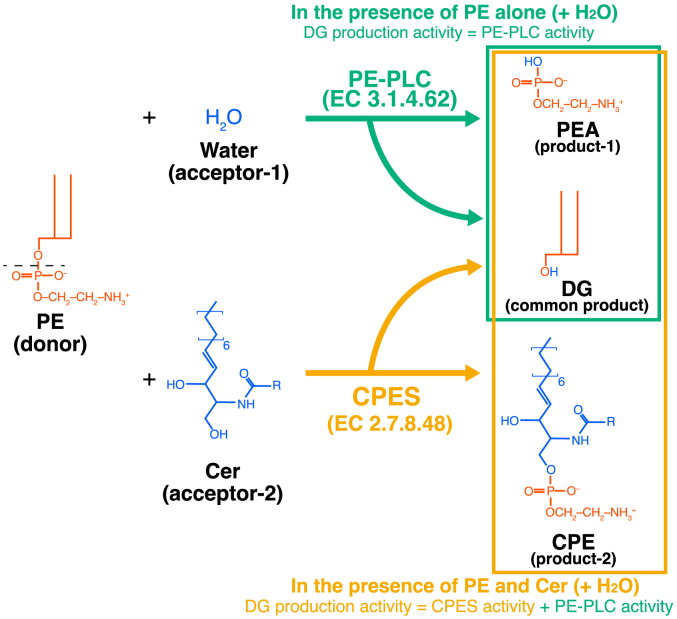
**Reactions of PE-PLC and CPES.** PE-PLC (EC 3.1.4.62) utilizes PE and water molecules to produce DG and PEA. CPES (EC 2.7.8.48) utilizes PE and Cer to produce DG and CPE. In the presence of PE, Cer, and water, SMS2 can exhibit both PE-PLC and CPES activities. Cer, ceramide; CPE, ceramide phosphoethanolamine; CPES, CPE synthase; DG, diacylglycerol; PE, phosphatidylethanolamine; PEA, phosphoethanolamine; PLC, phospholipase C; SMS, sphingomyelin synthase.

PLC catalyzes the hydrolysis of the linkage between glycerol and phosphate in glycerophospholipids. To date, 13 PLC isozymes have been identified in mammals, all of which exhibit PI(4,5)P_2_-specific PLC (PIP_2_-PLC) activity (EC 3.1.4.11) (18, 19). PC-specific PLC (PC-PLC; phosphatidylcholine choline phosphohydrolase) (EC 3.1.4.3) hydrolyzes PC to generate DG and phosphocholine (PCho) (Fig. 1). PE-specific PLC (PE-PLC) (EC 3.1.4.62) hydrolyzes PE to generate DG and phosphoethanolamine (PEA) (Fig. 2). SM synthase (SMS) (EC 2.7.8.27) cleaves PC to generate DG, followed by transfer of cleaved PCho to ceramide (Cer) and SM production (Fig. 1). CPE synthase (CPES) (EC 2.7.8.48) produces DG and CPE through cleavage of PE and transfer of cleaved PEA to Cer (Fig. 2). It is proposed that PLC, SMS, and CPES reactions proceed *via* a charge-relay system involving histidine and aspartate residues (Figs. 1 and 2 and Fig. S1) (18, 20, 21). The reactions are divided into two steps. The first step is a cleavage of glycerophospholipid (PC or PE, donor substrate) and the common product is DG (Figs. 1 and 2 and Fig. S1). In the second step, the cleaved polar head (PCho or PEA) is transferred to the acceptor (Figs. 1 and 2 and Fig. S1). The acceptors are different between PC-/PE-PLCs (acceptor: water molecule) and SMS/CPES (acceptor: Cer). Therefore, PC-PLC, PE-PLC, SMS, and CPES are different enzymatic reactions but produce the common product, DG.

PC-PLC and PE-PLC activities have been detected in a wide range of organisms (*e*.*g*., mammals, plants, and bacteria) and several plant and bacterial PC-/PE-PLC proteins have been cloned and characterized (Table 1). More than 70 years ago, PC-PLC activity was first detected in mammalian tissues such as the brain and liver using a radioisotope labeling technique (22, 23, 24, 25, 26, 27, 28). Moreover, PC-PLC activity is suggested to be related to several signal transduction pathways involved in inflammation, carcinogenesis, tumor progression, atherogenesis, and subarachnoid hemorrhage (29, 30, 31, 32). Membrane-associated PE-PLC activity in mammalian tissues was first observed approximately 35 years ago (16, 33, 34, 35, 36). However, mammalian PE-PLCs are yet to be identified. Unlike PI turnover-derived PI(4,5)P_2_, which mostly contains polyunsaturated fatty acids (PUFAs), primarily 20:4 (X:Y = total number of carbon atoms: total number of double bonds in the fatty acyl moiety of the glycerol backbone) at position *sn*-2, the major PC and PE molecular species in mammals primarily contain saturated fatty acids (SFAs) at position *sn-*1 and SFAs or monounsaturated fatty acids (MUFAs) at position *sn*-2 (37, 38, 39, 40, 41). Therefore, mammalian PC-PLC and PE-PLC primarily produce SFA- and/or MUFA-containing DG molecular species, whereas PIP_2_-PLCs produce mainly 20:4-containing DG molecular species.

**Table 1:** Comparison of bacterial, plant, and mammalian PLCs

Properties	Unknown mammalian PC-PLC	Unknown mammalian PE-PLC	Plant PLC (nonspecific PLC exhibiting PE-PLC and PC-PLC)	Bacterial PC-PLC	Identified mammalian PIP_2_-PLC
Subcellular location (the fraction containing PLC activity)	Detergent-insoluble fraction (47, 48, 49, 91) Nonraft domain in the plasma membrane (90) Cytosolic fraction (translocation from a perinuclear cytoplasmic area to the plasma membrane) (68)	Cell membrane (16, 35, 128) Cytosolic fraction (128)	Secreted (46) Cell membrane (soluble or membrane-associated proteins without apparent membrane-spanning domains) (46)	Soluble fraction (49, 129)	Cytosol (13, 130)
Effects of D609	Inhibition (57, 68, 90, 91)	Inhibition (16)	No effect (131)	Inhibition (58)	Unknown
Activator or inducer	phorbol ester (57) Triton X-100 (47) sodium deoxycholate (47)	phorbol ester and ethanol (16, 33) Prolactin (35)		sodium deoxycholate (93) divalent metal ion (Zn^2^, Ca^2+^, Mn^2+^, Mg^2+^) (93, 94)	Ca^2+^ (19) G protein-coupled receptor (GPCR) stimulation (132)
Inhibitor	D609 (57, 58, 59, 68, 90, 91)	Okadaic acid (protein phosphatase inhibitor) (33) Neomycin (128)	Elicitor (glycoprotein, cryptogein) (133) Al^3+^ (134) divalent metal ion (Zn^2+^, Co^2+^, Mn^2+^) (46, 92) No effect: Ca^2+^, Mg^2+^, EDTA, EGTA (46, 92)	EDTA (93, 94) D609 (58, 59)	U73122 (135)

PC, phosphatidylcholine; PE, phosphatidylethanolamine; PI(4,5)P2, phosphatidylinositol 4,5-bisphosphate; PLC, phospholipase C.

Plant and bacterial PC-PLCs have been discovered and cloned (42, 43, 44, 45, 46) (Table 1). However, neither the mammalian PC-PLC protein nor its corresponding genes have been identified (Table 1). The identified bacterial and plant PC-PLCs are soluble and secreted proteins, respectively (42, 43, 44, 45). In contrast, mammalian PC-PLC activity is associated with cell membranes, particularly detergent-resistant membrane (DRM) (47, 48, 49). Therefore, the identification of mammalian PC-PLC is hampered by difficulties in solubilizing and purifying PC-PLC proteins from mammalian tissues and in searching for mammalian PC-PLC genes based on identified bacterial and plant PC-PLC genes and mammalian PI-specific PLC genes, which are soluble proteins (13, 19). Therefore, to identify mammalian membrane–bound PC-PLC, an alternative approach beyond bioinformatics or the isolation of native PC-PLC from biological samples is required.

The mammalian SMS family consists of three isoforms, SMS1, SMS2, and SMS-related protein (SMSr), which contain a six times membrane-spanning core domain with a catalytic triad (His, His, and Asp), similar to the catalytic triad of LPPs (50). SMS1 and SMS2, but not SMSr, exhibit SMS activity. Moreover, SMS1, SMS2, and SMSr show CPES activity (51, 52, 53, 54). However, the detailed enzymological properties of mammalian SMS isoforms remain largely unknown because of the difficulty in acquiring highly purified proteins for enzyme activity assay *in vitro* (55).

Tricyclodecan-9-yl-xanthogenate (D609) has been known for its antiviral and antitumor properties (56). Because D609 showed inhibitory effects on phorbol ester–stimulated PC-PLC and PE-PLC in mammalian cells (16, 57) (Table 1), D609 has been widely used for characterization of the unidentified mammalian PC-/PE-PLC (58, 59). Because mammalian PC-PLC and PE-PLC genes and their proteins have been unidentified, the characterization of the PLCs has been limited to measuring enzyme activity using isotope labeling and inhibition of the enzymatic activity using D609. Interestingly, D609 was reported to inhibit not only PC-/PE-PLCs but also SMS (60) in mammalian cells.

We previously reported that PC-PLC activity was coimmunoprecipitated with DG kinase (DGK) δ in C2C12 cells, mouse myoblast cell line (61, 62, 63). Moreover, we showed that SMSr interacts with diacylglycerol kinase δ *via* their sterile α motif domains, protein–protein interaction modules (64). Notably, we recently demonstrated that highly purified SMSr displayed PAP, PI-PLC, PE-PLC, and PC-PLC activities in the absence of Cer (65). Moreover, the PC-PLC activity of SMSr was inhibited by D609, and SMSr preferentially hydrolyzed SFA-containing PC species compared to PUFA-containing PC species. Therefore, SMSr was the first mammalian membrane–associated protein, which possesses D609-sensitive PC-PLC activity, to be identified. Moreover, SMSr was the first mammalian PE-PLC to be identified.

Based on these results, we hypothesized that all SMS isoforms (SMS1, SMS2, and SMSr) possess PC-PLC and PE-PLC activities (donor: glycerophospholipid, acceptor: water) that are different from sphingolipid synthesis activity (donor: glycerophospholipid, acceptor: Cer) (Figs. 1 and 2). We recently demonstrated that purified SMS1 also displays PC-PLC and PE-PLC activities in the presence of glycerophospholipid and water, but not Cer. Moreover, SMS1 preferentially hydrolyzes SFA/MUFA-containing PC compared to PUFA-containing PC *in vitro* (54). Around the same time, another research group reported that SMS1 and SMS2 exhibited PC-PLC and SMS activities (66). However, this report is inconsistent with other reports by Kol *et al*. (53) and Ternes *et al*. (52), who stated that SMS2 displays dual activity as a CPES in addition to SMS activity in cells. Moreover, little is known about the detailed enzymological properties of SMS2, including PC acyl chain selectivity, D609 (a traditional PC-PLC inhibitor) sensitivity, and the catalytic site of SMS2 as a PLC.

In the present study, we evaluated the detailed enzymological properties of human SMS2 (NCBI Reference Sequence: NM_001136257.1, UniProt accession number: Q8NHU3) *in vitro* using a membrane protein purification system (54, 65), LC–MS/MS-based PLC/SMS/CPES activity measurement (49), and a detergent-free enzyme activity assay using SMS2-containing proteoliposomes prepared by dialysis (67) or using a cell-free protein expression system (53). Notably, highly purified SMS2 displayed PC-PLC, PE-PLC, and CPES activities, in addition to SMS activity, both in the presence (phospholipid-detergent mixed micelles) and absence (proteoliposomes, detergent-free near-native environments) of detergents. The PC-PLC activity of SMS2 was comparable to the SMS activity; however, the CPES and PE-PLC activities were lower than the SMS activity. SMS2 hydrolyzed MUFA-and/or SFA-containing PC and PE more preferentially than PUFA-containing PC and PE. The catalytic triad (His229, His272, and Asp276) of SMS2 (Fig. S1) is crucial for all the DG-producing activities. All DG-producing activities of SMS2 were inhibited by D609 and Zn^2+^. Intriguingly, DG inhibited the SMS activity of SMS2 (feedback control). Therefore, we propose that mammalian SMS2 is a candidate for long-sought mammalian PC- and PE-PLCs, which are membrane-associated, SFA/MUFA-containing PC and PE selective and D609 sensitive, and has unique enzymatic properties different from those of SMS1 and SMSr.

## Results

### Purification of SMS2 in the presence of detergents

**Figure 3:**
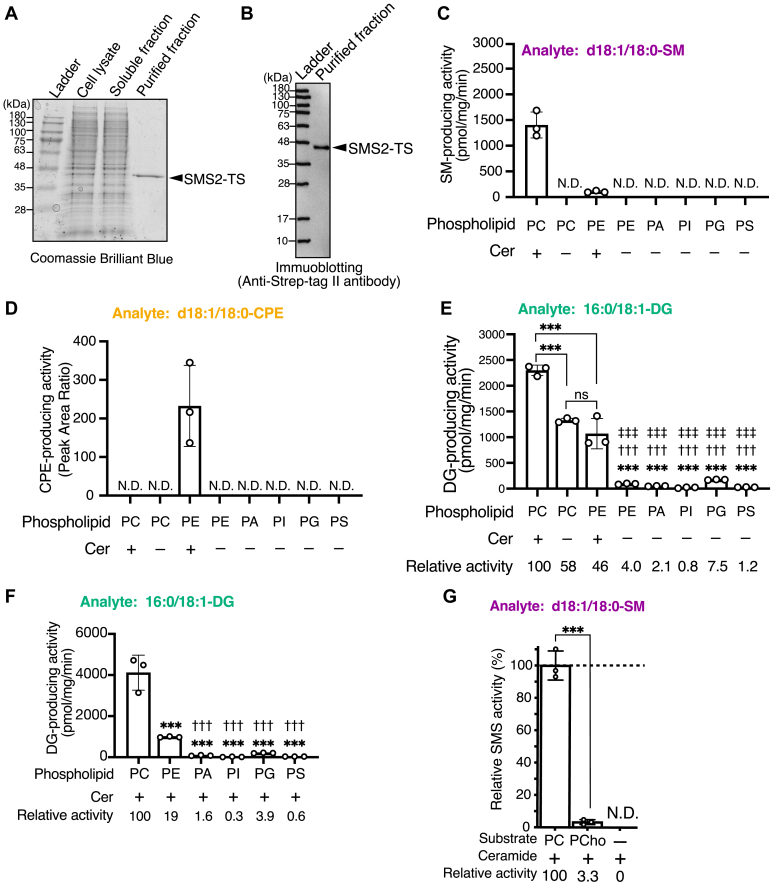
**Purification of SMS2 and PLC/SMS/CPES activity assay in the presence of detergents.***A* and *B*, C-terminal Twin-Strep–tagged human SMS2 (SMS2-TS) was expressed in HEK293 cells and purified by affinity chromatography using Strep-Tactin XT beads. Purified SMS-TS was analyzed using SDS-PAGE (12% gel), followed by Coomassie brilliant blue staining (*A*) or immunoblotting using an anti-Strep tag II antibody (1:1000 dilution in Can Get Signal Solution 1 (Toyobo)). BlueStar Prestained Protein Ladder (#NE-MWP03, Nippon Genetics) was used for molecular mass markers. *C–E*, DG-, SM-, and CPE-producing activities of SMS2-TS, which was purified in the presence of detergents, were measured using LC–MS/MS. d18:1/18:0-Cer and/or 16:0/18:1-phospholipids (PC, PE, PA, PI, PG, or PS) in the detergent mixed micelles were used as substrates. Purified SMS2-TS (10 μl (approximately 500 ng protein) of the sample) was used for the enzyme assays. The peak area ratio of 16:0/18:1-DG (*C*), d18:1/18:0-SM (*D*), and d18:1/18:0-CPE (*E*) in the samples were calculated using an internal standard (I.S.) (0.2 ng/μl of 15:0/18:1-DG, d18:0/12:0-SM, and d18:1/24:0-CPE). Purified SMS2-TS contained detectable 16:0/18:1-DG, d18:1/18:0-SM that probably came from HEK293 cell membranes. We calculated DG-producing activity of SMS2-TS after subtracting these backgrounds. The absolute quantities of 16:0/18:1-DG and d18:1/18:0-SM in the samples were determined from calibration curves generated using commercially available purified lipids (16:0/18:1-DG and d18:1/18:0-SM) (Fig. S2). The micelles contained detectable 16:0/18:1-DG, which was likely contaminated by the phospholipid samples. Therefore, we calculated DG-generating activity after subtracting these backgrounds (the purified SMS2 mixed with the micelle without incubation at 37 °C for 2 h). The values are presented as the mean ± SD (n = 3, technical replicates). ND, not detectable. ns, not significant, ∗∗∗, *p* < 0.005 (*versus* PC/Cer); ^†††^, *p* < 0.005 (*versus* PC without Cer); ^‡‡‡^, *p* < 0.005 (*versus* PE/Cer). One-way ANOVA with Tukey–Kramer’s post hoc test was used. *F*, the DG-generating activity of SMS2-TS in the presence of Cer and phospholipids was measured using LC–MS/MS. The micelles containing d18:1/18:0-Cer and 16:0/18:1-glycerophospholipid (50 μM each) were incubated with purified SMS2. The values are presented as the mean ± SD (n = 3, technical replicates). ∗∗∗, *p* < 0.005 (*versus* PC/Cer); ^†††^, *p* < 0.005 (*versus* PE/Cer). One-way ANOVA with Tukey’s post hoc test was used. *G*, SMS activity of SMS2-TS in the presence of Cer/PC or Cer/phosphocholine (*PCho*) was measured using LC–MS/MS. The micelles containing d18:1/18:0-Cer and 16:0/18:1-PC (50 μM each) or PCho (50 μM each) was incubated with purified SMS2 (approximately 500 ng). The SMS activity in the samples was determined by the quantitation of d18:1/18:0-SM using LC–MS/MS. Values are presented as percentages of the SMS activity in the presence of PC and Cer (set to 100%). Values are presented as the mean ± SD (n = 3 technical replicates). ND, not detectable. ∗∗∗, *p* < 0.005 (PCho/Cer *versus* PC/Cer). Student’s *t* test was used. Cer, ceramide; CPE, ceramide phosphoethanolamine; CPES, ceramide phosphoethanolamine synthase; DG, diacylglycerol; PA, phosphatidic acid; PC, phosphatidylcholine; PE, phosphatidylethanolamine; PG, phosphatidylglycerol; PI, phosphatidylinositol; PLC, phospholipase C; PS, phosphatidylserine; SM, sphingomyelin; SMS, sphingomyelin synthase.

Since human SMS2 is a six-transmembrane protein (50), we chose mammalian cell expression and an efficient one-step purification system using a Twin-Strep (TS)-tag and Strep-Tactin XT (54) to purify human SMS2. C-terminal TS-tagged human WT SMS2 (SMS2-TS) was overexpressed in HEK293 cells and purified by affinity chromatography using Strep-Tactin XT beads in the presence of detergent (Fig. 3, *A* and *B*). Approximately 50 μg of SMS2-TS was purified from 16 of 150-mm dishes (approximately 3.2 × 10^8^ cells). A single band with a molecular mass of approximately 46 kDa was detected by Coomassie brilliant blue staining (Fig. 3*A*) and immunoblotting with an anti-Strep II tag antibody after SDS-PAGE (Fig. 3*B*). The calculated molecular masses of SMS2-TS were 46.6 kDa (SMS2, 42.3 kDa; Tobacco Etch Virus (TEV) site and TS-Tag (4.3 kDa)). These results indicate that SMS2-TS were obtained with high purity.

All enzyme activity assays were performed in a detergent-based micelle environment (Figs. 3 and Figure 6, Figure 7, Figure 8, Figure 9, Figure 10, Figure 11, Figure 12, Figure 13) except for the activity assays using SMS2-TS reconstituted in the detergent-free proteoliposomes (Fig. 4) and SMS2-TS expressed by the cell-free expression system in the presence of liposomes (detergent-free) (Fig. 5).

**Figure 4:**
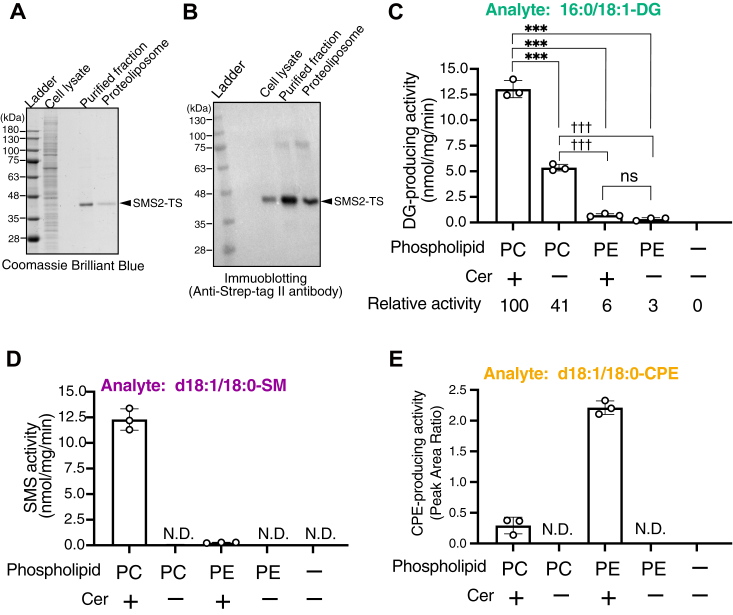
**SMS/PC-PLC activities of SMS2-TS in proteoliposomes in the absence of detergents.***A* and *B*, SMS2-TS was expressed in HEK293 cells and purified using affinity chromatography with Strep-Tactin XT beads in the presence of detergents. Purified SMS-TS was reconstituted into the liposomes (18:1/18:1-PC). The SMS2 containing liposomes (detergent-free) were subjected to SDS-PAGE (12% gel), followed by Coomassie brilliant blue staining (*A*) and immunoblotting (*B*). *C–E*, quantification of 16:0/18:1-DG (*C*), d18:1/18:0-SM (*D*) and d18:1/18:0-CPE (*E*) generated by SMS2-TS containing liposomes (detergent-free) when incubated with 16:0/18:1-PC or PE and/or d18:1/18:0-Cer for 2 h at 37 °C. The detected intensity of the analyte in the sample was quantified using an internal standard (I.S.) (0.2 ng/μl of 15:0/18:1-DG, d18:0/12:0-SM, or d18:1/24:0-CPE). Values are presented as the mean ± SD (n = 3, technical replicates). ND, not detectable. ns, not significant, ∗∗∗, *p* < 0.005 (*versus* PC/Cer); ^†††^, *p* < 0.005 (*versus* PC without Cer). One-way ANOVA with Tukey’s post hoc test was used. Cer, ceramide; CPE, ceramide phosphoethanolamine; DG, diacylglycerol; PC, phosphatidylcholine; PE, phosphatidylethanolamine; PLC, phospholipase C; SM, sphingomyelin; SMS, sphingomyelin synthase; TS, Twin-Strep.

**Figure 5:**
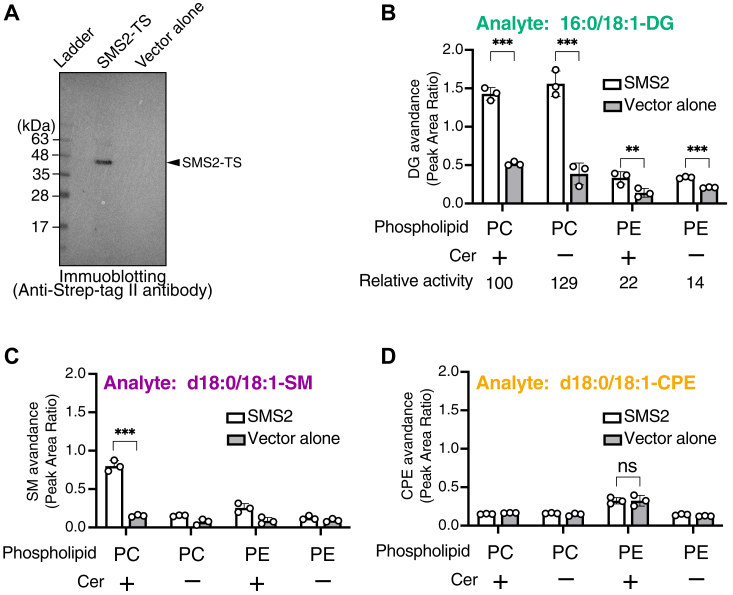
**Cell-free expression of SMS2-TS in the presence of proteoliposomes and its PLC/SMS/CPES activities in the absence of detergents.***A*, SMS2-TS was expressed using the human cell-free protein expression system (detergent-free) and reconstituted in the presence of liposomes (18:1/18:1-PC, detergent-free). After translation reactions with or without SMS2-TS–containing plasmid, the SMS2-TS protein in the sample was detected using immunoblotting with anti-Strep tag II antibody. BlueStar Prestained Protein Ladder was used for molecular weight markers. An empty vector (vector alone) was used for the expression system as a control. *B–D*, quantification of 16:0/18:1-DG (*B*), d18:1/18:0-SM (*C*), and d18:1/18:0-CPE (*D*) generated through SMS2-TS expressed by the cell-free expression system. After expression, the sample containing SMS2-TS or negative control (vector alone) was incubated with 16:0/18:1-glycerophospholipids and/or d18:0/18:1-Cer for 16 h at 37 °C. The detected intensity of the analyte in the sample was quantified using an internal standard (I.S.) (0.2 ng/μl of 15:0/18:1-DG, d18:0/12:0-SM, or d18:1/24:0-CPE). The vector alone samples contained detectable 16:0/18:1-DG, d18:1/18:0-SM, and d18:1/18:0-CPE that probably came from cell-free expression mixtures or lipid solutions. To determine whether SMS2-TS exhibits the activities, we compared the DG, SM, and CPE levels of SMS2-TS expressing samples to that of vector alone. Values are presented as the mean ± SD (n = 3, technical replicates). ∗∗, *p* < 0.01; ∗∗∗, *p* < 0.005; ns, not significant. Student’s *t* test was used. Cer, ceramide; CPE, ceramide phosphoethanolamine; CPES, CPE synthase; DG, diacylglycerol; PC, phosphatidylcholine; PLC, phospholipase C; SM, sphingomyelin; SMS, sphingomyelin synthase; TS, Twin-Strep.

**Figure 6:**
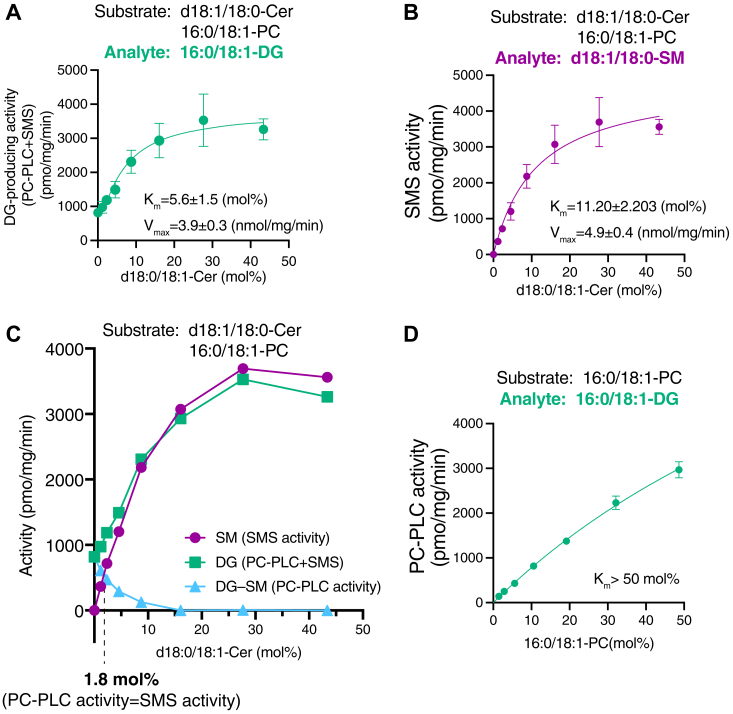
**Effects of Cer and PC concentrations on SMS/PC-PLC activities of SMS2.***A* and *B*, 16:0/18:1-DG (*A*) and d18:1/18:0-SM (*B*) generated by SMS2-TS, which was purified in the presence of detergents, were analyzed in the presence of 50 μM of 16:0/18:1-PC and various concentrations of d18:1/18:0-Cer in detergent mixed micelles as indicated. The detected intensity of the analyte in the sample was quantified using an internal standard (I.S.) (0.2 ng/μl of 15:0/18:1-DG, d18:0/12:0-SM, and d18:1/24:0-SM). The absolute quantities of 16:0/18:1-DG and d18:1/18:0-SM were determined from calibration curves generated using commercially available lipids. Values are presented as the mean ± SD (n = 3, technical replicates). *C*, 16:0/18:1-DG–generating activity (PC-PLC activity + SMS activity) (*A*), d18:1/18:0-SM–generating activity (SMS activity) (*B*), and PC-PLC activity (subtracted values (16:0/18:1-DG – d18:1/18:0-SM)) of SMS2-TS were plotted. *D*, enzyme kinetic analysis of purified SMS2 (PC-PLC) with 16:0/18:1-PC mixed micelles. DG-generating activity (PC-PLC activity) was plotted as a function of the 16:0/18:1-PC concentration in the mixed micelles (mol%). Values represent averages of triplicate measurements. Data are represented as the mean ± SD. Cer, ceramide; DG, diacylglycerol; PC, phosphatidylcholine; PLC, phospholipase C; SM, sphingomyelin; SMS, sphingomyelin synthase; TS, Twin-Strep.

**Figure 7:**
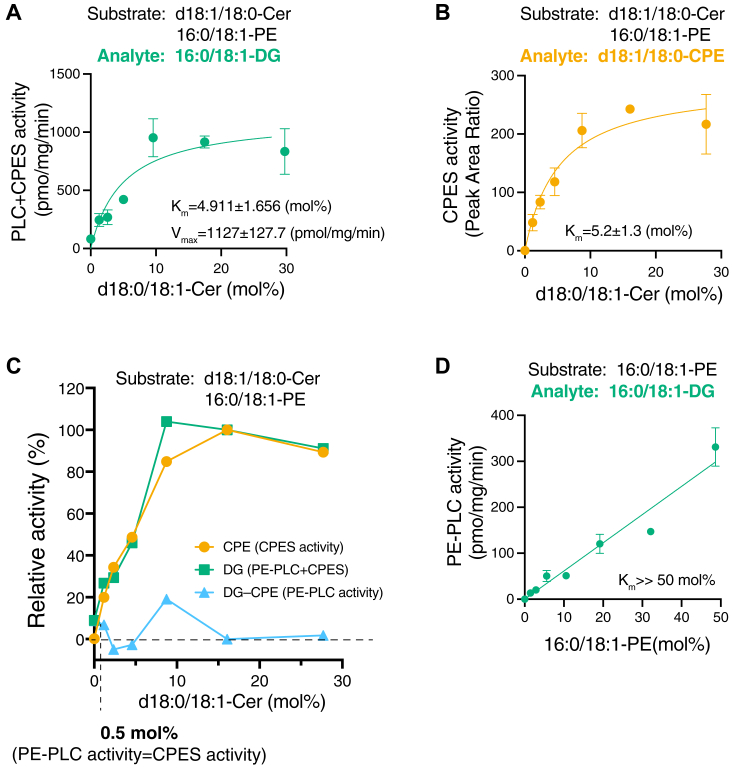
**Effects of Cer and PE concentrations on CPES/PE-PLC activities of SMS2.***A* and *B*, 16:0/18:1-DG (*A*) and d18:1/18:0-CPE (*B*) generated by SMS2-TS, which was purified in the presence of detergents, were analyzed in the presence of 50 μM of 16:0/18:1-PE and various concentrations of d18:1/18:0-Cer in detergent mixed micelles as indicated. The detected intensity of the analyte in the sample was quantified using an internal standard (I.S.) (0.2 ng/μl of 15:0/18:1-DG, d18:0/12:0-SM, and d18:1/24:0-CPE). The absolute quantities of 16:0/18:1-DG were determined based on calibration curves generated using commercially available lipids. The values are presented as the mean ± SD (n = 3, technical replicates). *C*, relative 16:0/18:1-DG- and d18:1/18:0-CPE-generating activities of SMS2-TS (A and B). The subtracted values (relative 16:0/18:1-DG – relative d18:1/18:0-CPE) were also plotted as the relative PE-PLC activity of SMS2-TS. *D*, enzyme kinetic analysis of purified SMS2 (PE-PLC) with 16:0/18:1-PE. DG-generating activity (PE-PLC activity) was plotted as a function of the 16:0/18:1-PE concentration in the mixed micelles (mol%). Values represent averages of triplicate measurements. Data are presented as the mean ± SD (n = 3, technical replicates). Cer, ceramide; CPE synthase; CPE, ceramide phosphoethanolamine; CPES, DG, diacylglycerol; PE, phosphatidylethanolamine; PLC, phospholipase C; SM, sphingomyelin; SMS, sphingomyelin synthase; TS, Twin-Strep.

**Figure 8:**
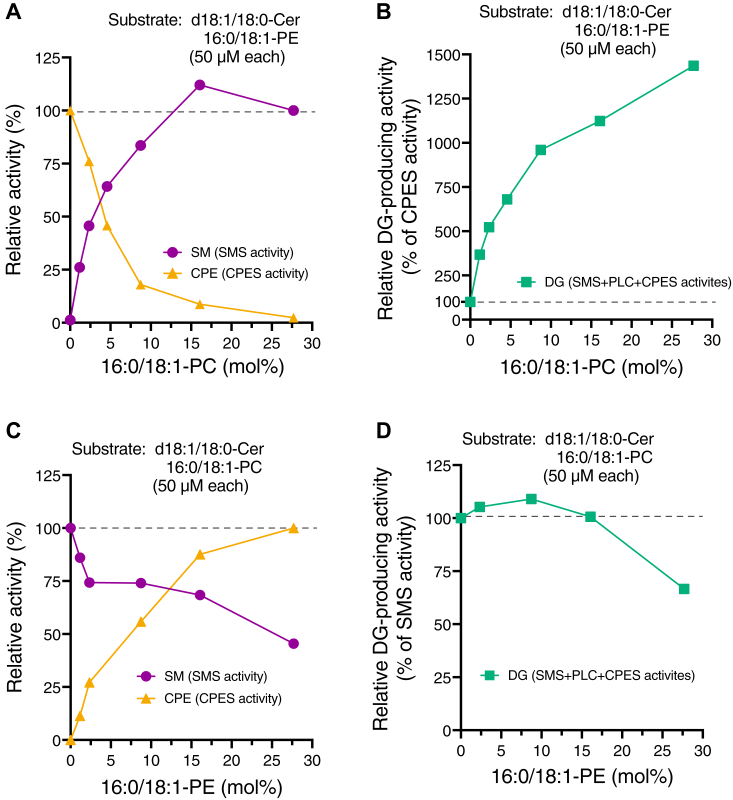
**Effects of PC and PE concentrations on PLC/SMS/CPES activities of SMS2.***A* and *B*, the effect of PC concentrations on the enzyme activities of SMS2, which was purified in the presence of detergents, was determined. Relative d18:1/18:0-CPE- and d18:1/18:0-SM–producing activities (*A*) and relative DG-producing activities (*B*) of SMS2-TS were analyzed in the presence of 50 μM each of 16:0/18:1-PE and d18:1/18:0-Cer, and various concentrations of 16:0/18:1-PC in mixed micelles. *C* and *D*, the effect of PE concentrations on the enzyme activities of SMS2, which was purified in the presence of detergents, was determined. Relative d18:1/18:0-CPE- and d18:1/18:0-SM–producing activities (*C*) and relative DG-producing activities (*D*) of SMS2-TS were analyzed in the presence of 50 μM each of 16:0/18:1-PC, d18:1/18:0-Cer, and various concentrations of 16:0/18:1-PE in detergent mixed micelles. The DG, SM, and CPE produced by SMS2 were quantified using LC–MS/MS. Data are represented as the mean ± SD (n = 3, technical replicates). Cer, ceramide; CPE, ceramide phosphoethanolamine; CPES, CPE synthase; DG, diacylglycerol; PC, phosphatidylcholine; PE, phosphatidylethanolamine; PLC, phospholipase C; SM, sphingomyelin; SMS, sphingomyelin synthase; TS, Twin-Strep.

**Figure 9:**
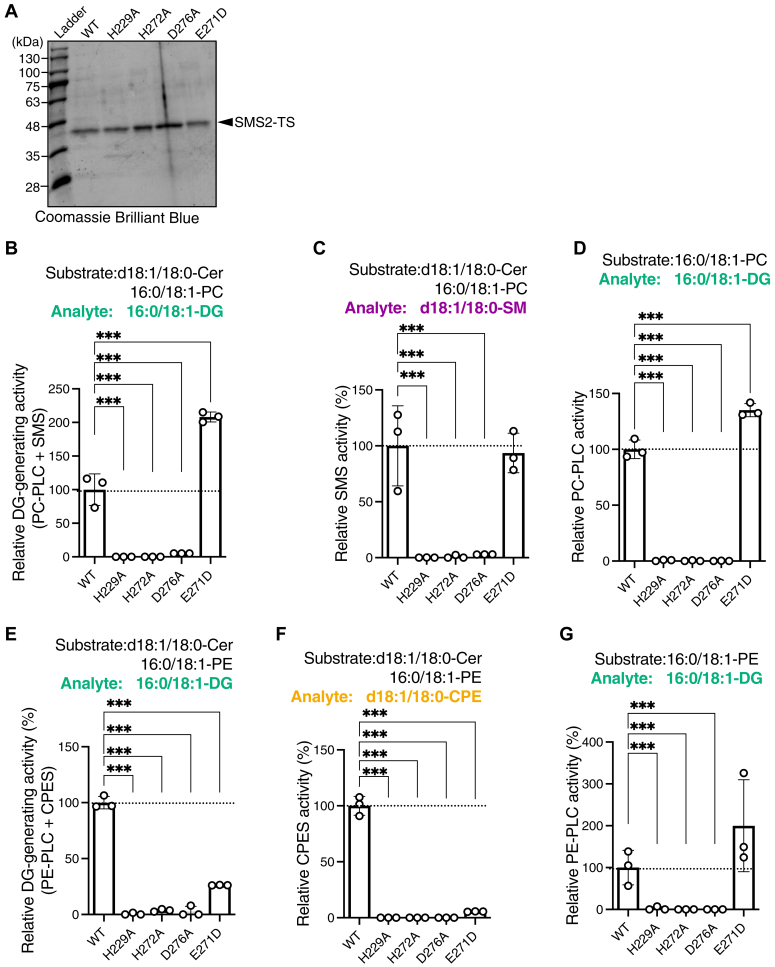
**Effects of the mutations of the conserved catalytic triad of SMS2 on PLC/SMS/CPES activities.***A*, WT human SMS2-TS (WT), SMS2 point-mutated in catalytic triad (H229A, H272A, or D276A) (50), and SMS2 point-mutated in loop b (E271D), which is crucial for CPES activity of SMS2 (53), were expressed in HEK293 cells and purified in the presence of DDM/CHS detergents. Purified proteins were detected using SDS-PAGE followed by Coomassie brilliant blue staining. BlueStar Prestained Protein Ladder was used for molecular weight markers. *B*–*G*, the effects of the point-mutations on SMS activities (analyte: DG (*B*) and SM (*C*)), PC-PLC activity (*D*), CPES activity (analyte: DG (*E*) and CPE (*F*)), and PE-PLC activity (*G*) of SMS2. Purified proteins (approximately 500 ng) were incubated with 16:0/18:1-PC, 16:0/18:1-PE, and/or d18:1/18:0-Cer in detergent mixed micelles for 2 h at 37 °C. The DG-, SM-, and CPE-producing activities were determined using LC–MS/MS and normalized based on the protein concentration of samples. The relative activity was standardized through comparison to the WT SMS2 (set at 100%). The data are represented as the mean ± SD (n = 3, technical replicates). ∗∗, *p* < 0.01 and ∗∗∗, *p* < 0.005. One-way ANOVA with Dunnett’s post hoc test was used. Cer, ceramide; CHS, cholesteryl hemisuccinate; CPE, ceramide phosphoethanolamine; CPES, CPE synthase; DDM, n-dodecyl-β-D-maltoside; DG, diacylglycerol; PC, phosphatidylcholine; PE, phosphatidylethanolamine; PLC, phospholipase C; SM, sphingomyelin; SMS, sphingomyelin synthase; TS, Twin-Strep.

**Figure 10:**
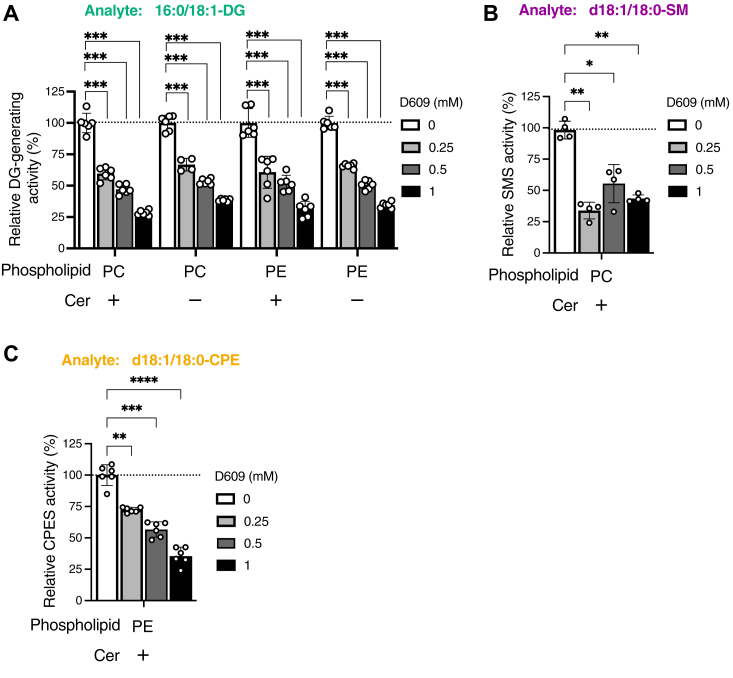
**Effects of D609, a PC-PLC/SMS inhibitor, on PC-PLC, PE-PLC, SMS, and CPES activities of SMS2.***A*, DG-generating activities of SMS2, which was purified in the presence of detergents, were analyzed in the presence of various concentrations of D609 (0, 0.25, 0.5, and 1 mM). Purified WT SMS2-TS (approximately 500 ng) was incubated in the presence of 16:0/18:1-PC alone, 16:0/18:1-PC and d18:1/18:0-Cer, 16:0/18:1-PE alone, or 16:0/18:1-PE and d18:1/18:0-Cer–containing detergent mixed micelles for 2 h at 37 °C. Produced 16:0/18:1-DG was quantified using LC–MS/MS. Values are presented as percentages of the activity of SMS2 in the absence of D609 (set at 100%). Values are presented as the mean ± SD (n = 6, technical replicates). ∗∗∗, *p* < 0.005 (*versus* control (without D609)). One-way ANOVA with Dunnett’s post hoc test was used. *B*, SM-producing activities of SMS2, which was purified in the presence of detergents, in the presence of 16:0/18:1-PC and d18:1/18:0-Cer–containing detergent mixed micelles were analyzed in the presence of various concentrations of D609 (0, 0.25, 0.5, and 1 mM). Values are presented as percentages of the SMS activity of SMS2 in the absence of D609 (set to 100%). The values are presented as the mean ± SD (n = 4, technical replicates). ∗, *p* < 0.05; ∗∗, *p* < 0.01 (*versus* control (without D609)). One-way ANOVA with Dunnett’s post hoc test was used. *C*, CPE-producing activities of SMS2, which was purified in the presence of detergents, in the presence of 16:0/18:1-PE and d18:1/18:0-Cer–containing detergent mixed micelles were analyzed in the presence of various concentrations of D609 (0, 0.25, 0.5, and 1 mM). Values are presented as percentages of the CPES activity of SMS2 in the absence of D609 (set at 100%). Values are presented as the mean ± SD (n = 6, technical replicates). ∗∗, *p* < 0.01; ∗∗∗, *p* < 0.005 (*versus* control (without D609)). One-way ANOVA with Dunnett’s post hoc test was used. Cer, ceramide; CPE, ceramide phosphoethanolamine; CPES, CPE synthase; DG, diacylglycerol; PC, phosphatidylcholine; PE, phosphatidylethanolamine; PLC, phospholipase C; SM, sphingomyelin; SMS, sphingomyelin synthase; TS, Twin-Strep.

**Figure 11:**
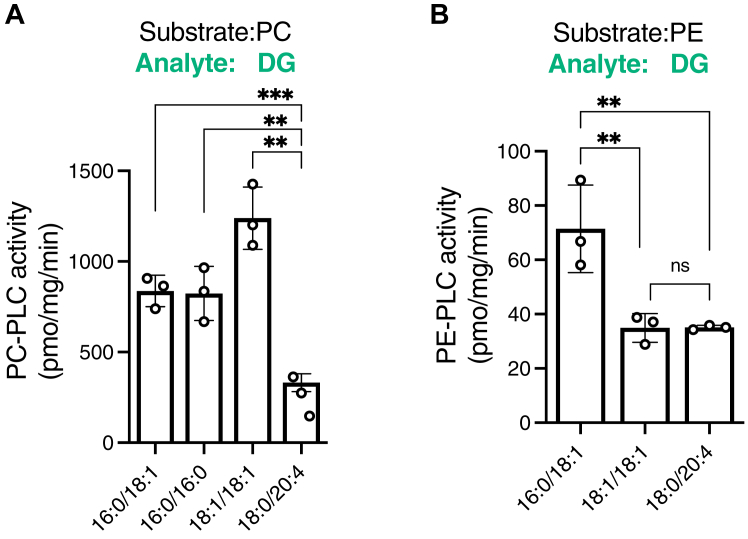
**Effects of acyl chains of PC and PE on PC- and PE-PLC activities of SMS2.** Comparison of the enzymatic activities of SMS2, which was purified in the presence of detergents, with PC species (16:0/18:1, 16:0/16:0, 18:1/18:1, and 18:0/20:4-PC) (*A*) and PE species (16:0/18:1, 18:1/18:1, and 18:0/20:4-PE) (*B*) as substrates. Purified SMS2-TS (approximately 500 ng) was mixed with PC- or PE-detergent mixed micelles (50 μM of phospholipids) and incubated for 2 h at 37 °C. The DG species produced in the samples were quantified using LC–MS/MS. The absolute quantities of 16:0/18:1-, 16:0/16:0-, 18:1/18:1-, and 18:0/20:4-DG in the samples were determined from the calibration curves generated using commercially available lipids (16:0/18:1-DG, 16:0/16:0-DG, 18:1/18:1-DG, and 18:0/20:4-DG). Values are presented as the mean ± SD (n = 3, technical replicates). ∗∗, *p* < 0.01; ∗∗∗, *p* < 0.001; ns, not significant. One-way ANOVA with Tukey’s *post hoc* test. Cer, ceramide; CPE, ceramide phosphoethanolamine; CPES, CPE synthase; DG, diacylglycerol; PC, phosphatidylcholine; PE, phosphatidylethanolamine; PLC, phospholipase C; SM, sphingomyelin; SMS, sphingomyelin synthase; TS, Twin-Strep.

**Figure 12:**
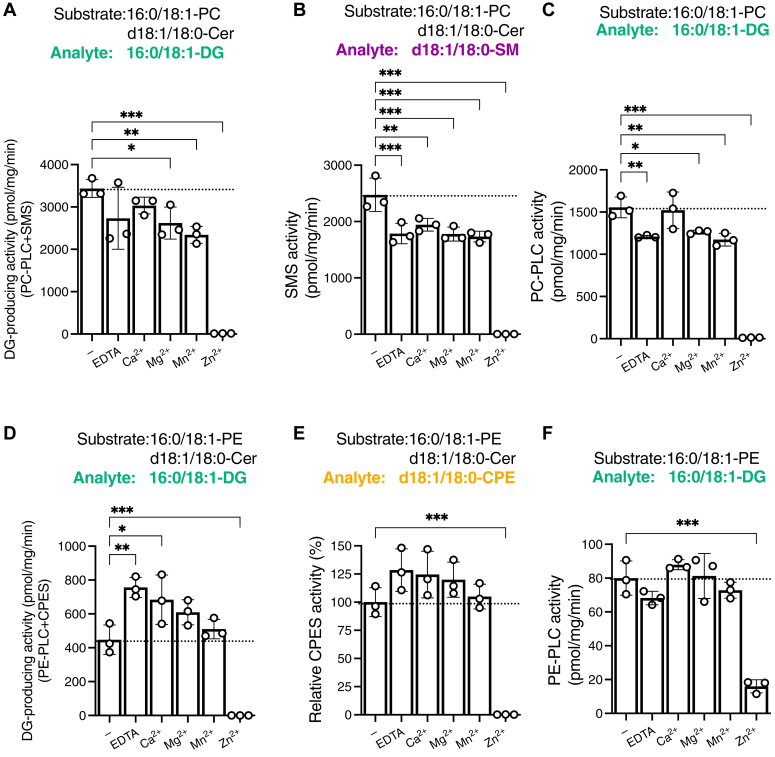
**Effects of metal ions on the PLC/SMS/CPES activities of SMS2.***A–F* the effects of several metal ions (500 μM), Mg^2+^, Ca^2+^, Mn^2+^ or Zn^2+^, or EDTA (500 μM) on SMS (analyte: 16:0/18:1-DG (*A*) and d18:1/18:0-SM (*B*)), PC-PLC (*C*), CPES (analyte: 16:0/18:1-DG (*D*) and d18:1/18:0-CPE (E)), and PE-PLC (*F*) activities of SMS2, which was purified in the presence of detergents, in the presence of mixed micelles (50 μM lipids) were determined using LC–MS/MS. The absolute quantities of 16:0/18:1-DG and d18:1/18:0-SM in the samples were determined based on the calibration curves. Values are presented as the mean ± SD (n = 3, technical replicates). ∗, *p* < 0.05; ∗∗, *p* < 0.01; and ∗∗∗, *p* < 0.001 (*versus* control (without metal ions)). One-way ANOVA with Dunnett’s post hoc test was used. Cer, ceramide; CPE, ceramide phosphoethanolamine; CPES, CPE synthase; DG, diacylglycerol; PC, phosphatidylcholine; PE, phosphatidylethanolamine; PLC, phospholipase C; SM, sphingomyelin; SMS, sphingomyelin synthase; TS, Twin-Strep.

**Figure 13:**
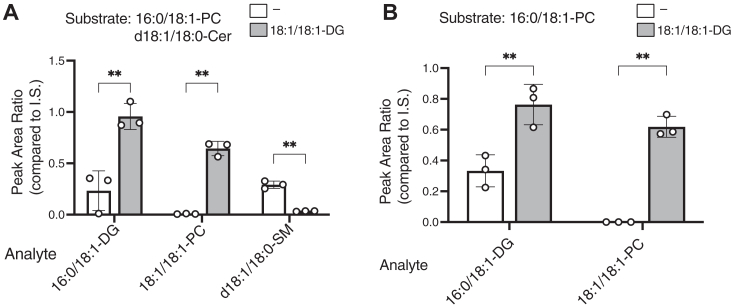
**Effects of 18:1/18:1-DG on PC-PLC and SMS activities of SMS2.** SMS2 was purified in the presence of detergents. SMS activity (substrates: 16:0/18:1-PC and d18:0/18:1-Cer detergent mixed micelles) (*A*) and PC-PLC activity (substrate: 16:0/18:1-PC detergent mixed micelles) (*B*) were determined in the presence or absence of 18:0/18:1-DG (16 mol%). The detected intensity of the analyte in the sample was determined using an internal standard (I.S.) 0.2 ng/μl of 15:0/18:1-DG, d18:0/12:0-SM, and 15:0/18:1-PC. Values are presented as the mean ± SD (n = 3, technical replicates). ∗∗, *p* < 0.01 (*vs*. control (without 18:1/18:1-DG)). Student’s *t* test was used. Cer, ceramide; CPE, ceramide phosphoethanolamine; CPES, CPE synthase; DG, diacylglycerol; PC, phosphatidylcholine; PLC, phospholipase C; SM, sphingomyelin; SMS, sphingomyelin synthase; TS, Twin-Strep.

### Enzymological characterization of SMS2 in the presence of detergents using SMS2 purified in the presence of detergents

LC–MS/MS offers advantages such as high-sensitivity, high-throughput, radioisotope-free, direct detection of analytes, and reliable quantification compared to other analytical methods (*e*.*g*.*,* those using radioisotope labeling and TLC (47) and indirect fluorescent PLC activity assay (68)). Thus, we adopted LC–MS/MS-based PLC activity assay (49, 54, 64) for characterization of SMS2 *in vitro*. The amounts of detected lipids were normalized by calculating the ratio of the response for the internal standard (I.S.) and the analyte (peak area ratio), enabling relative quantification (69). For absolute quantification, we generated calibration curves using commercially available purified lipids (16:0/18:1-DG and d18:1/18:0-SM) (Fig. S2), as detailed in Experimental procedure.

At first, to test whether purified SMS2-TS retained SMS and CPES activities (52, 53), we evaluated SM- and CPE-producing activities of SMS2-TS in the presence of detergents, 16:0/18:1-glycerophospholipid (PC, PE, PA, PI, phosphatidylserine (PS), or phosphatidylglycerol (PG)), and/or d18:1/18:0-Cer-mixed micelles (Fig. 3, *C* and *D*). SMS2 produced SM in the presence of PC and Cer-containing micelles (Fig. 3*C*). CPE was generated in the presence of SMS2, PE, and Cer (Fig. 3*D*). These results indicate that purified SMS2-TS retained its SMS and CPES activities and that the activities can be detected in the LC-MS/MS–based enzyme activity assay.

Next, we tested whether purified SMS2-TS exhibits PC-PLC and PE-PLC activities (PC- and PE-dependent DG-producing activities in the absence of Cer) in the presence of detergents (Fig. 3*E*). SMS2 showed strong and weak DG-producing activities in the presence of PC and PE, respectively (Fig. 3*E*). We compared the absolute DG-producing activities (pmol of DG/mg protein/min) (Fig. 3*E*) and SM-producing activities (pmol of SM/mg protein/min) (Fig. 3*C*), which were calculated using standard curves (Fig. S2). In the presence of Cer/PC mixed micelles and water, SM-producing activity of SMS2 was approximately 1350 pmol/mg/min (Fig. 3*C*), whereas DG-producing activity was approximately 2300 pmol/mg/min (Fig. 3*E*), which is approximately 1.7-fold stronger than the SM-producing activity. In the presence of only PC mixed micelles and water (PC alone, without Cer), the DG-producing activity (PC-PLC activity) was approximately 1300 pmol/mg/min (Fig. 3*E*). Thus, the sum of the SM producing activity and the DG-producing activity in the presence of PC alone (PC-PLC activity) is nearly equal to the DG-producing activity in the presence of PC and Cer. These results strongly suggest that SMS2 exhibits dual enzyme activity (SMS and PC-PLC activities) in the presence of PC (donor), Cer (acceptor-1), and water (acceptor-2) (Fig. 1). Compared with the DG-producing activity of SMS2 in the presence of PC, Cer, and water (SMS + PC-PLC activity), SMS2 showed substantial PC-PLC activity (approximately 58% of SMS + PC-PLC activity), considerable CPES + PE-PLC activity (approximately 46% of SMS + PC-PLC activity) in the presence of PE, Cer, and water (Fig. 3*E*), and detectable PE-PLC activity (approximately 4% of SMS + PC-PLC activity) in the presence of PE and water (Fig. 3*E*).

In addition to PC- and PE-PLC activities, SMS2 also exhibited PG-PLC activity (approximately 7.5% of SMS + PC-PLC activity) (Fig. 3*E*). However, SMS2 produces only trace amounts of DG in the presence of PA, PI, and PS (Fig. 3*E*). Thus, we focused on PC and PE as substrates for SMS2 for further analysis (Figure 4, Figure 5, Figure 6, Figure 7, Figure 8, Figure 9, Figure 10, Figure 11, Figure 12, Figure 13).

Because Cer enhanced the DG-producing activity of SMS2 in the presence of PC or PE (Fig. 3*E*), we tested whether Cer affected the activities of other DG-producing activities (Fig. 3*F*). Other DG-generating activities (PAP, PI-PLC, PG-PLC, and PS-PLC) were not increased by Cer, suggesting that SMS2 possesses phosphotransferase activity only toward PC and PE.

It has been proposed that the DG-producing reaction of SMS can be divided into two steps: step 1: PC hydrolyzation (production of PCho and DG), and step 2: transfer of PCho to Cer (20) (Fig. 1 and Fig. S1). Thus, we tested whether free PCho, but not PC, could be used for SM synthesis by SMS2 (Fig. 3*G*). However, SM synthesis activity in the presence of PCho and Cer was considerably weaker than that of SMS activity (approximately 3.3%) (Fig. 3*G*), indicating that PC is the major substrate and is necessary for the SMS activity of SMS2.

### Enzymological characterization of SMS2 under near-native environments (in the absence of detergents) using SMS2 purified in the presence of detergents

As shown in Figure 3, we purified SMS2 in the presence of detergents and measured its activity using detergents-containing mixed micelles. Detergents may render the catalytically active site of SMS2 leaky for water and consequently affecting the enzymatic properties of SMS2. To evaluate the enzymological characterization of SMS2 in near-native environments, detergents in the purified SMS2-TS samples (Fig. 3, *A* and *B*) were removed by dialysis and the enzyme was reconstituted in detergent-free 1,2-dioleoyl-sn-glycero-3-PCho (18:1/18:1-PC) liposomes. Using the proteoliposome containing SMS2-TS (approximately 25 ng of protein/sample), the SM-, CPE-, and DG-producing activities were measured by LC–MS/MS quantification of DG, SM, and CPE (Fig. 4). After reconstitution, purified SMS2-TS (approximately 46 kDa) in the proteoliposomes was clearly detected using Coomassie brilliant blue and anti-Strep-Tag II antibody after SDS-PAGE (Fig. 4, *A* and *B*).

The DG- and SM-producing activities of SMS2 were detected in the presence of 16:0/18:1-PC and d18:1/18:0-Cer–containing liposomes (Fig. 4, *C* and *D*), indicating that SMS2 was active in proteoliposomes. Intriguingly, the specific SMS activity of SMS2 in proteoliposomes was approximately 10-fold stronger (approximately 12 nmol SM/mg protein/min (Fig. 4*C*)) than the activity in micelles (approximately 1.35 nmol SM/mg protein/min (Fig. 3*C*)). As shown in Figure 4*E*, SMS and CPES activities of SMS2 were clearly detected. DG-generating activities were detected in the presence of PC or PE with or without Cer, indicating that SMS2 displays multiple enzymatic activities (SMS, CPE, PC-PLC, and PE-PLC) in detergent-free proteoliposomes, near native environments (Fig. 4*C*), as detected using detergent mixed micellar assays (Fig. 3). Compared to the DG-producing activity of SMS2 in the presence of 16:0/18:1-PC and d18:0/18:1-Cer (substrates for SMS + PC-PLC activity), SMS2 showed substantial PC-PLC activity (approximately 41% of SMS + PC-PLC activity) in the presence of PC alone, weak CPES activity (approximately 6% of SMS + PC-PLC activity) in the presence of PE and Cer, and low PE-PLC activity (approximately 3% of SMS + PC-PLC activity) in the presence of PE alone (Fig. 4*C*). In the proteoliposomes, the CPES and PE-PLC activities of SMS2 were nearly equal (Fig. 4*C*). Compared to the relative CPES activity of SMS2 in the presence of detergents (46% of SMS + PC-PLC activity) (Fig. 3*E*), that of SMS2 in proteoliposomes was substantially weaker (approximately 6% of SMS + PC-PLC activity) (Fig. 4*C*). These results indicated that the substrate selectivity of the phosphotransferase activity of SMS2 for PE is reduced in proteoliposomes, implying that SMS2 primarily displays SMS and PC-PLC activities in cell membranes.

### Enzymological characterization of SMS2 under near-native environments (in the absence of detergents) using SMS2 expressed in a detergent-free human cell-free protein expression system

To further evaluate the enzymatic properties of SMS2 in the absence of detergents, we used a detergent-free human cell-free protein expression system. In this system, pT7-IRES DNA served as the expression vector and SMS2-TS cDNA was subcloned into the vector. An empty vector was used as a negative control. In the cell-free system, SMS2-TS was expressed in the presence of 18:1/18:1-PC liposomes to generate proteoliposomes. As shown in Figure 5*A*, SMS2-TS (approximately 46 kDa) was strongly expressed and was detected by immunoblotting using an anti-Strep-Tag II antibody.

We measured SM-, CPE-, and DG-producing activities in detergent-free proteoliposomes with or without SMS2-TS expressed by the cell-free expression system (Fig. 5, *B* and *C*). As shown in Figure 5*B*, SMS2-TS showed DG-producing activity in the presence of PC alone, PE alone, PC/Cer, and PE/Cer, indicating that SMS2 possesses PC-PLC and PE-PLC activities, in addition to SMS and CPES activities, under completely detergent-free conditions (near-native environments). Moreover, SMS2-TS exhibited SM-producing activity in the presence of PC and Cer (Fig. 5*C*), indicating that SMS2 retains its SMS activity. However, we were unable to detect the CPE produced by SMS2 in this experiment (Fig. 5*D*). The detected intensity of d18:0/18:1-SM in the samples (Fig. 5*C*) was approximately 10-fold lower than that in samples containing SMS2-proteoliposomes reconstituted from detergent-containing samples (Fig. 4*B*). Therefore, the CPES activity was lower than the limit of detection (Fig. 5*D*).

### Effects of Cer on the PC-PLC and PE-PLC activities of purified SMS2 in the presence of detergents

Next, we analyzed the effects of the Cer concentration on the PC-PLC and SMS activities of SMS2 in the presence of detergents (Fig. 6). The DG-producing activity of SMS2 in the presence of PC increased in a Cer-dependent manner (Fig. 6*A*). The PC-PLC activity (DG-generating activity in the absence of Cer) was 25.0% of the maximum DG-producing activity (SMS activity + PC-PLC activity) of SMS2 in the presence of 45.9 mol% of Cer (Fig. 6*A*). SM-producing activity also increased in a Cer-dependent manner (Fig. 6*B*). To compare the SMS activity/PC-PLC activity ratio, the relative PC-PLC activity (DG-producing activity (SMS activity + PC-PLC activity (Fig. 6A))—SM-producing activity (Fig. 6*B*)) is shown in Figure 6*C*. We confirmed that the maximum SM-producing activity was almost the same as the maximum DG-producing activity (Fig. 6*C*). PC-PLC–dependent DG-producing activities decreased in a Cer-dependent manner (Fig. 6*C*). Notably, in the presence of approximately 2 mol% Cer and 4 mol% PC (1:2 ratio), SMS activity was almost equal to PC-PLC activity (Fig. 6*C*).

The Michaelis–Menten constant (*K*_m_) of SMS2 as a PLC for PC was determined (Fig. 6*D*). PC-PLC activity increased in a PC-dependent manner (Fig. 6*D*). However, we could not determine *K*_m_ (>50 mol%) or *V*_max_ (>3000 pmol/min/mg protein).

Next, we examined the effects of the Cer concentration on the PE-PLC and CPES activities of SMS2 (Fig. 7). DG-producing activity of SMS2 in the presence of PE was augmented in a Cer-dependent manner (Fig. 7*A*). The PE-PLC activity (DG-generating activity in the absence of Cer) was 8.9% of the maximum DG-producing activity (CPES activity + PE-PLC activity) of SMS2 in the presence of 29.7% of Cer (Fig. 7*A*). CPE-producing activity also increased in a Cer-dependent manner (Fig. 7*B*). To compare the CPES activity/PE-PLC activity ratio, the relative PE-PLC activity (DG-producing activity (Fig. 7*A*: CPES activity + PE-PLC activity)—relative CPE-producing activity (Fig. 7*B*)) is shown in Figure 7*C*. PE-PLC–dependent DG-producing activities essentially decreased in a Cer-dependent manner (Fig. 7*C*). As shown in Figure 7*C*, in the presence of approximately 0.5 mol% Cer and 4.5 mol% PE (1:9 ratio), CPES activity is almost equal to PE-PLC activity (Fig. 7*C*).

The Michaelis–Menten constant (*K*_m_) of SMS2 for PE was determined (Fig. 7*D*). PE-PLC activity increased in a PE-dependent manner (Fig. 7*D*). However, we failed to determine *K*_m_ (>50 mol%) nor *V*_max_ (>300 pmol/min/mg protein).

### Effect of PC concentration on CPES activity of purified SMS2 and effect of PE concentration on SMS activity of purified SMS2 in the presence of detergents

Next, we evaluated competition between PC and PE as SMS2 substrates in the presence of Cer (Fig. 8). Interestingly, CPE-producing activity in the presence of PE and Cer dramatically decreased in a PC concentration–dependent manner (Fig. 8*A*), whereas SM-producing activity (Fig. 8*A*) and DG-producing activity (Fig. 8*B*) substantially increased in a PC-dependent manner. The relative SMS activity of SMS2 surpassed 50% of the maximum SMS activity (in the presence of approximately 27 mol% PC and 5 mol% PE (5:1 ratio)) in the presence of approximately 5 mol% PC and PE (1:1 ratio) (Fig. 8*A*), and the CPES activity of SMS2 was reduced to less than 50% of the maximum CPES activity (in the absence of PC) in the presence of approximately 5 mol% PC and PE (1:1 ratio).

As shown in Figure 8*C*, the SM-producing activity of SMS2 in the presence of PC and Cer decreased slightly in a PE-dependent manner. Nevertheless, even in the presence of approximately 28 mol% PE and 5 mol% PC (6:1 ratio), the SMS activity was maintained at approximately 50% of the maximum activity (observed in the absence of PE) (Fig. 8*C*). Moreover, DG-producing activity slightly decreased in a PE-dependent manner (Fig. 8*D*). These results indicate that SMS2 preferentially utilizes PC rather than PE as a substrate in the presence of Cer.

### Effects of point mutations of catalytic triad of purified SMS2 on its SMS/CPES/PC-PLC/PE-PLC activities in the presence of detergents

Huitema *et al*. reported that SMS isoforms contain motifs D3 (C-G-D-X_3_-S-G-**H**-T) and D4 (**H**-Y-T-X-**D**-V-X_3_-Y-X_6_-F-X_2_-Y-H), which form a catalytic triad (bold/underlined fonts in D3 and D4 motifs: H229, H272, and D276 in human SMS2), mediating nucleophilic attack on the lipid phosphate ester bond (50) (Fig. S1). The catalytic triad is crucial for SMS activity (51, 70). Kol *et al*. reported that a Glu residue adjacent to the catalytic histidine in D4 (E271 in human SMS2) is crucial for the CPES activity of SMS2 (53). Thus, we evaluated the effects of point mutations in the catalytic triad (H229, H272, and D276) and E271 on the SMS, CPES, PC-PLC, and PE-PLC activities of SMS2. Using purified WT SMS2-TS and its mutant proteins (SMS2^H229A^-TS, SMS2^H272A^-TS, SMS2^D276A^-TS, and SMS2^E271D^-TS) (Fig. 9*A*), we measured the SMS, CPES, PC-PLC, and PE-PLC activities of the proteins (Fig. 9, *B*–*G*). Substitution of the catalytic triad (H229A, H272A, and H276A) with Ala abolished the SMS activity (Fig. 9, *B* and *C*), PC-PLC activity (Fig. 9, *B* and *D*), PE-PLC activity (Fig. 9, *E* and *G*), and CPES activity (Fig. 9, *E* and *F*). The SMS2 with E271D mutation showed lower CPES activity (Fig. 9, *E* and *F*), but no significant change was observed in PE-PLC activity (Fig. 9*G*) or SMS activity (Fig. 9*C*). Interestingly, the PC-PLC activity of SMS2^E271D^ was higher than that of WT (Fig. 9*D*).

### Effects of D609 on PC-PLC/PE-PLC/SMS/CPES activities of purified SMS2 in the presence of detergents

D609 is a well-known inhibitor of PC-PLC, SMS, and PE-PLC (16, 58, 59, 60). Recently, we demonstrated that the SMS activity of purified SMS1, but not its PC-PLC activity, is inhibited by D609 (54). Moreover, the PAP and PC-PLC activities of purified SMSr were attenuated by D609 (65). These findings led us to evaluate the effects of D609 on PC-PLC, PE-PLC, SMS, and CPES activities of SMS2 (Fig. 10). In the presence of PC alone, PE alone, PC/Cer and PE/Cer, DG-generating activities of SMS2 were significantly decreased in a D609-dependent manner (250 μM, 500 μM, and 1 mM of D609) (Fig. 10*A*). SM-producing (Fig. 10*B*) and CPE-producing (Fig. 10*D*) activities were also decreased by D609. These results indicated that D609 inhibited all enzyme activities of SMS2 (PC-PLC, PE-PLC, SMS, and CPES).

### PC and PE species selectivity of PC- and PE-PLC activities of purified SMS2 in the presence of detergents

We analyzed the effects of several PC species (16:0/18:1-PC, 16:0/16:0-PC, 18:1/18:1-PC, and 18:0/20:4-PC) as substrates on the PC-PLC activity of SMS2 (Fig. 11*A*). In the presence of MUFA-containing PC (18:1/18:1-PC), SFA-containing PC (16:0/16:0-PC), and SFA-/MUFA-containing PC (16:0/18:1-PC), SMS2 showed strong PC-PLC activity (Fig. 11*A*). In the presence of PUFA-containing PC (18:0/20:4-PC), SMS2 exhibited weak PC-PLC activity, which was approximately 30% of the maximum PC-PLC activity observed in the presence of 18:1/18:1-PC (Fig. 11*A*). These results suggest that SMS2 preferentially hydrolyzes the SFA- and/or MUFA-containing PC species.

The effects of the PE molecular species (16:0/18:1-PE, 18:1/18:1/PE, and 18:0/20:4-PE) on the PE-PLC activity of SMS2 were also analyzed (Fig. 11*B*). In the presence of PE containing both MUFA and SFA (16:0/18:1-PE), SMS2 showed the highest PE-PLC activity. Compared with 16:0/18:1-PE, SMS2 exhibited approximately 50% PE-PLC activity in the presence of 18:1/18:1-PE or 18:0/20:4-PE (Fig. 11*B*). The PE-PLC activity of SMS2 in the presence of 16:0/16:0-PE was not quantitatively detected, for unknown reasons. These results suggested that SMS2 preferentially hydrolyzes 16:0/18:1-PE (SFA- and MUFA-containing PE species).

### Effects of metal ions on PC-PLC/PE-PLC/SMS/CPES activities of purified SMS2 in the presence of detergents

We previously showed that Zn^2+^ inhibits DG-producing activities of both SMS1 and SMSr (54). These findings led us to evaluate the effects of divalent metal ions on SMS2. We analyzed the effects of several divalent metal ions (500 μM), Ca^2+^, Mg^2+^, Mn^2+^ and Zn^2+^ on PC-PLC, PE-PLC, SMS, and CPES activities of SMS2 (Fig. 12). All activities (SMS, PC-PLC, PE-PLC, and CPES) of SMS2 were strongly inhibited by Zn^2+^ (Fig. 12, *A*–*F*). The SM-producing activity of SMS2 was slightly inhibited by EDTA, Ca^2+^, Mg^2+^, or Mn^2+^ (Fig. 12*B*). PC-PLC activity modestly decreased in the presence of EDTA, Ca^2+^, or Mn^2+^ (Fig. 12*C*).

### Effects of DG on SMS activities of purified SMS2 in the presence of detergents

Hanada *et al*. reported that DG, but not triacylglycerol, strongly inhibits SMS activity in homogenates of CHO-K1 cells (71). In addition, Huitema *et al*. reported that SMS can transfer PCho from PC and SM to DG (the reverse reaction of SMS) (50). Therefore, we determined whether 18:1/18:1-DG, a DG species different from 16:0/18:1-DG derived from 16:0/18:1-PC, affected the SM and DG production activities of purified SMS2 in the presence of 16:0/18:1-PC and d18:1/18:0-Cer as its substrates (Fig. 13*A*). 18:1/18:1-DG inhibited the SMS activity (Fig. 13*A*). The purified SMS2 produced 18:1/18:1-PC in the presence of 18:1/18:1-DG (Fig. 13*A*). In the presence of 16:0/18:1-PC alone, 18:1/18:1-DG enhanced the production of 16:0/18:1-DG by SMS2 (PC-PLC activity and PC:DG choline phosphotransferase activity), and 18:1/18:1-PC was also generated (Fig. 13*B*). These results suggest that SMS2 exhibits PC:DG choline phosphotransferase activity that is competitive with SMS activity and that DG competitively inhibits SMS activity by PC:DG choline phosphotransferase activity.

## Discussion

DG production by SMS2 has been observed in the plasma membrane (72) and Golgi apparatus (73). It was thought without any doubt that DG production by SMS2 originated from the SM production process (Cer-dependent DG production) (Fig. 1). Recently, we showed that SMS1 and SMSr exhibit PC-PLC and PE-PLC activities in the absence of Cer (51, 55). These finding led us to hypothesize that SMS2 possesses PC-PLC and PE-PLC activities (Figs. 1 and 2, and Fig. S1). In the present study, we highly purified human SMS2 from mammalian cells (Fig. 3). Using purified SMS2 and LC-MS/MS–based enzyme activity assay (49), we demonstrated that SMS2 acts as a multifunctional enzyme with SMS, PC-PLC, CPES, and PE-PLC activities. Chiang *et al*. recently reported that SMS2 displays PC-PLC and SMS activities (66). Moreover, Kol *et al*. (53) and Ternes *et al*. (52) reported that SMS2 displays dual activity as a CPES in addition to SMS. These reports support the findings of the present study.

Our main new findings are as follows (1): The accessibility of water molecules to the catalytic region of SMS2 can be altered by detergent. However, highly purified SMS2 showed PC-PLC and PE-PLC activities under detergent-free near-native environments (Figure 3, Figure 4, Figure 5 and Table 2) (2). SMS, CPES, PC-PLC, and PE-PLC activities of SMS2 were quantitatively compared with each other (Figure 3, Figure 4, Figure 5, Figure 6, Figure 7 and Table 2) (3). The enzymatic parameters (*K*_m_ and *V*_max_) of SMS, CPES, PC-PLC, and PE-PLC activities were determined (Figure 6, Figure 7 and Table 2) (4). SMS and PC-/PE-PLC activities were competitive each other (Figs. 6 and 7 and Table 2). In the presence of approximately 2 mol% Cer and 4 mol% PC (1:2 ratio), PC-PLC activity was almost equal to SMS activity (Fig. 6) (5). Competition analysis between PC and PE as SMS2 substrates in the presence of Cer revealed that SMS2 preferentially utilizes PC rather than PE as a substrate in the presence of Cer (Fig. 8 and Table 2) (6). The catalytic triad (His229, His272, and Asp276) of SMS2 is crucial for PC-PLC, PE-PLC, and CPES activities in addition to SMS activity (Fig. 9 and Table 2) (7). All enzymological activities of SMS2 (SMS/CPES/PC-PLC/PE-PLC) were inhibited by D609 (a traditional PC-PLC inhibitor) (Fig. 10 and Table 2) (8). SMS2 as PC-/PE-PLC showed substrate selectivity for saturated fatty acid– and/or MUFA-containing PC and PE species (Fig. 11 and Table 2) (9). All enzymological activities of SMS2 (SMS/CPES/PC-PLC/PE-PLC) were inhibited by Zn^2+^ (Fig. 12 and Table 2) (10). DG competitively inhibited SMS activity of SMS2 *via* PC:DG choline phosphotransferase activity (Fig. 13 and Table 2) (11). Mammalian SMS2 was proposed to be a candidate for long-sought mammalian PC- and PE-PLCs, which are membrane-associated, SFA/MUFA-containing PC and PE-selective and D609-sensitive (Figs. 10 and 11 and Tables 1 and 2).

**Table 2:** Comparison of SMS2 (our findings and previous reports), SMS1, and SMSr

Properties	SMS2 (our findings)	SMS2 (previous reports)	SMS1	SMSr
Subcellular localization		Plasma membrane (70, 84, 103, 104) Medial/trans Golgi apparatus (70, 84, 103, 104)	Medial/trans Golgi apparatus (70, 84, 103, 104)	Endoplasmic reticulum (ER) (51, 103, 104)
Location of catalytic triad (Fig. 14 and Fig. S1)		Plasma membrane: cell surface (outer leaflet) (50) Golgi: lumen (136)	Golgi apparatus: lumen (50, 136)	ER: lumen (51, 137)
Reaction				
Assay condition	In the presence of detergents: SMS, CPES, PC-PLC, and PE-PLC Reconstitution of proteoliposomes: SMS, CPES, PC-PLC, and PE-PLC Cell-free expression system: SMS, PC-PLC, PE-PLC	In the presence of detergents: SMS and PC-PLC (66) Cell-free expression system: SMS and CPES (53)	In the presence of detergents: SMS, CPES, PE-PLC, and PC-PLC (54) Cell-free expression system: SMS (53)	In the presence of detergents: CPES, PE-PLC, PC-PLC, PI-PLC, PIP_2_-PLC, PG-PLC, and PAP (65) Cell-free expression system: CPES (53)
Substrates	*SMS activity***:** Cer + PC *CPES***:** Cer + PE *PC-PLC*: *PC + water* *PE-PLC*: PE + water	*SMS activity***:**Cer + PC (50) *CPES****:*** Cer + PE *PC-PLC****:*** PC + water	*SMS activity* (50) Cer + PC *CPES activity***:** Cer + PE (54, 138) *Phospholipid-hydrolysis activity***:** PC (54, 66) PE (54)	*CPES activity* (51) Cer + PE *Phospholipid-hydrolysis activity* (65)**:** PA PI > PIP_2_ PE (102, 139) PC PG
Products (Lipids)	*SMS activity***:** DG + SM *CPES activity***:** DG + CPE *PC-PLC activity***:** DG + PCho *PE-PLC activity***:** DG + PEA	*SMS activity***:** DG + SM *CPES activity***:** DG + CPE *PC-PLC activity***:** DG + PCho	*SMS activity***:** DG + SM *CPES activity***:** DG + CPE *Phospholipid-hydrolysis activity***:** DG + polar head	*CPES activity***:** DG + CPE *Phospholipid-hydrolysis activity***:** DG + polar head
Relative DG-producing activity	Mixed-micelle activity assay**:** PC/Cer > PC > PE/Cer > PE 100 58 46 4 Proteoliposome and liposome activity assay**:** PC/Cer > PC > PE/Cer > PE 100 41 6 3	*Unknown*	Mixed-micelle activity assay (54)**:** PC/Cer > PC > PE/Cer > PE 100 36 23 4	Mixed-micelle activity assay (65)**:** PA > PI > PE > PC > PG > Cer/PE 100 3.6 1.2 0.9 0.4 0.3
Kinetic parameter (*K*_m_) in mixed-micelle activity assay	*SMS activity(d18*:*1/18*:*0-Cer)***:** 11.2 mol% *CPES activity(d18*:*1/18*:*0-Cer*)**:** 5.1 mol% *PC-PLC activity(16*:*0/18*:*1-PC)***:** > 50 mol% *PE-PLC activity(16*:*0/18*:*1-PC)***:** > 50 mol%	SMS activity (16:0–06:0 NBD PC)**:** 104 μM (66) PC-PLC activity (16:0–06:0 NBD PC)**:** 57 μM (66)	*SMS activity(d18*:*1/18*:*0-Cer)* (54) 4.5 mol% PC-PLC activity (16:018:1-PC) (54)**:** > 30 mol%	PAP activity (16:0/18:1-PA) (65)**:** 3.9 mol% PI-PLC activity *(16*:*0/18*:*1-PI)* (65)**:** 6.8 mol%
Maximum rate of reaction (*V*_max_) in mixed-micelle activity assay (pmol/mg/min)	*SMS activity(d18*:*1/18*:*0-Cer)***:** SM production: 4850 DG production: 3910 *CPES activity(d18*:*1/18*:*0-Cer)* DG production: 1127 *PC-PLC activity(16*:*0/18*:*1-PC)***:** >3000 *PE-PLC activity(16*:*018*:*1-PC)**:*** >300	*Unknown (Reported data are in arbitrary units)*	*SMS activity(d18*:*1/18*:*0-Cer and 16*:*0/18*:*1-PC)* (54)**:** SM production: 240 DG production: 288 *PC-PLC activity(16*:*0/18*:*1-PC)* (54)**:** > 200	*PAP activity(16*:*0/18*:*1-PA)* (65)**:** 704 *PI-PLC activity(16*:*0/18*:*1-PI)* (65)**:** 32
Acyl chain specificity (*in vitro* study)	SFA/MUFA-containing PC SFA-containing PE	*Unknown*	SFA/MUFA-containing PC (54)	SFA/MUFA-containing PC (65) SFA/MUFA-containing PA (65)
Sensitivity to inhibitor: D609	*SMS activity*: Inhibition *CPES activity*: Inhibition *PC-PLC activity*: Inhibition *PE-PLC activity*: Inhibition	*SMS activity*: Inhibition (60, 140) *CPES*: Unknown *PC-PLC*: Unknown	*SMS activity***:** Inhibition (50) *PC-PLC activity*: No effect (54)	*PAP activity***:** Inhibition (65) *PI-PLC activity***:** No effect (65) *PC-PLC activity***:** Inhibition (65)
Effects of 18:1/18:1-DG	*SMS activity*: Inhibition *PC-PLC activity*: Promotion (indirectly)	*SMS activity***:** Inhibition (71)	*SMS activity*: Inhibition (54) *CPES activity*: Inhibition (54)	*Unknown*
Effects of metal ions	*SMS activity***:** Inhibition (strongly): Zn^2+^ Inhibition (slightly):Mg^2+^, Ca^2+^, Mn^2+^, EDTA *CPES activity***:** Inhibition (strongly): Zn^2+^: No effect: Ca^2+^, Mg^2+^, Mn^2+^, EDTA *PC-PLC activity***:** Inhibition (strongly): Zn^2+^ Inhibition (slightly): Mg^2+^, Mn^2+^, EDTA No effect: Ca^2+^ *PE-PLC activity***:** Inhibition (strongly): Zn^2+^: No effect: Ca^2+^, Mg^2+^, Mn^2+^, EDTA	*Unknown*	*SMS activity* (54)**:** Zn^2+^: Inhibition No effect: :Mg^2+^, Ca^2+^, Mn^2+^ *PC-PLC activity* (54)**:** Inhibition: Mn^2+^, Zn^2+^ No effect: Mg^2+^, Ca^2+^	*PAP activity* (54)**:** Mg^2+^: Inhibition Ca^2+^: No effect Mn^2+^: No effect Zn^2+^: Inhibition *PI-PLC activity* (54)**:** Mg^2+^: Inhibition Ca^2+^: Inhibition Mn^2+^: No effect Zn^2+^: Inhibition
The mutation of amino acid residues related to catalytic activity	*SMS activity***:** *Catalytic triad* (H229A, H272A, D276A) CPES activity**:** E271D Catalytic triad (H229A, H272A, D276A) PC-PLC activity**:** Catalytic triad (H229A, H272A, D276A) *PE-PLC activity****:*** Catalytic triad (H229A, H272A, D276A)	*SMS activity***:** *Catalytic triad* (H229A, H272A, D276A) (70) C188S (136) C221S (136) CPES activity**:** E271D (53)	*SMS activity***:** *Catalytic triad* (H285A, H328A, D332A) (70) S283A (70) C227S, C244S, C277S, C311S, C321S (136)	CPES**:** Catalytic triad (H301A, H344A, D348E) (51, 102) E205D, C293A, G294A, D295A (102) PAP (65): D348E PI-PLC (65)**:** D348E

Cer, ceramide; CPES, ceramide phosphoethanolamine synthase; DG, diacylglycerol; MUFA, monounsaturated fatty acid; PAP, PA phosphatase; PC, phosphatidylcholine; PCho, phosphocholine; PE, phosphatidylethanolamine; PEA, phosphoethanolamine; PG, phosphatidylglycerol; PI(4,5)P_2_, phosphatidylinositol 4,5-bisphosphate; PI, phosphatidylinositol; PLC, phospholipase C; SFA, saturated fatty acid; SMS, sphingomyelin synthase.

There are limitations in distinguishing between SMS and PC-PLC activities of SMS2 *in vivo*. For example, these two reactions utilize the same substrate (PC) and produce the same product (DG) (Fig. 1). Moreover, although there are several reports demonstrating that SM levels are decreased *in vivo* using SMS2-KO mice (74, 75, 76, 77, 78, 79, 80, 81), DG levels were not significantly decreased by SMS2-KO (82, 83). Thus, we focused the enzymological properties of SMS2 *in vitro* instead of *in vivo* to address our hypothesis. However, further studies will be required to elucidate the biological significance of DG produced by the PC-PLC activity of SMS2 *in vivo*.

Because the accessibility of water molecules to the catalytic region of SMS2 can be altered by detergent, it is possible that the detergent-based micelle activity assay does not always reflect the enzyme activities of membrane proteins in the native environments. However, we demonstrated that SMS2 exhibits PC-PLC, PE-PLC, and CPES activities both in the presence (phospholipid-detergent mixed micelles) and absence (proteoliposomes, detergent-free near-native environments) of detergents (Figure 3, Figure 4, Figure 5). Intriguingly, there were dissimilarities between the enzymological properties of SMS2 with detergents (Fig. 3) and those of SMS2 under near-native environments with detergent-free proteoliposomes (Figs. 4 and 5). For example, the relative CPES activity of SMS2 in proteoliposomes (approximately 6% of SMS activity (Fig. 4*C*)) was lower than that observed under detergent-containing environments (approximately 46% of SMS activity (Fig. 3*C*)). This discrepancy may be because the detergent altered the environments of the catalytic site in SMS2, making it more accessible to Cer in the presence of PE.

**Figure 14:**
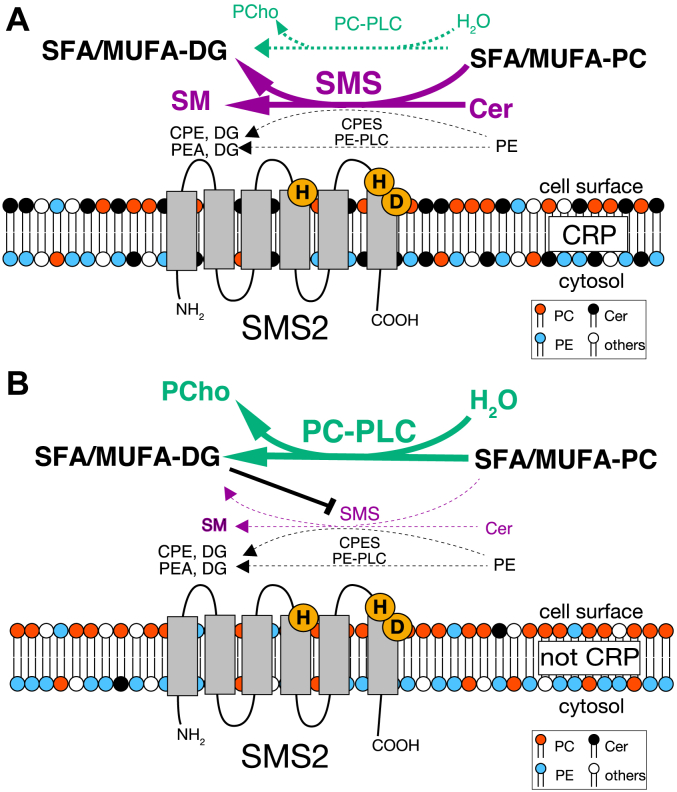
**Schematic model for the function of SMS2 as a DG-producing enzyme in the plasma membrane.** Human SMS2 is primarily localized to the plasma membrane (84). SMS2 is partially localized to the DRM (74). Cer is concentrated in DRM (CRP at least 5 mol% (88)). The catalytic triad of SMS2 is located in exoplasmic leaflets (*green circles*). PC, especially SFA- and/or MUFA-containing PC, is enriched in the exoplasmic leaflets of the plasma membrane bilayer, whereas PE is enriched in the cytosolic leaflets (141). Consequently, SMS2 may produce SFA- and/or MUFA-DG and SM primarily through its SMS activity and secondarily through PC-PLC activity in CRP because the SMS activity of SMS2 surpassed its PC-PLC activity in the presence of approximately 2 mol% Cer and 4 mol% PC (Fig. 6*C*). *A*, in the plasma membrane regions, except for CRP (*B*), SMS2 may produce SFA- and/or MUFA-DG primarily through PC-PLC activity because SMS2 preferentially hydrolyzes SFA- and/or MUFA-containing DG (Fig. 11*A*). Moreover, DG enhances PC-PLC activity and inhibits SMS activity because the PC:DG choline phosphotransferase activity of SMS2 competes with its SMS activity (Fig. 13*A*). Therefore, SMS2 may synthesize only trace amounts of SM in the membrane regions, with the exception of CRPs (*B*). Cer, ceramide; CPE, ceramide phosphoethanolamine; CPES, CPE synthase; CRP, Cer-rich platform; DG, diacylglycerol; DRM, detergent-resistant membrane; MUFA, monounsaturated fatty acid; PC, phosphatidylcholine; PC, phosphatidylcholine; PE, phosphatidylethanolamine; PLC, phospholipase C; SFA, SFA, saturated fatty acid; SM, sphingomyelin; SMS, sphingomyelin synthase; TS, Twin-Strep.

Which enzyme activity between SMS/PC-PLC and CPES/PE-PLC would SMS2 primarily exhibit *in vivo*? Human SMS2 is primarily localized in the plasma membrane and partly in the Golgi apparatus (84). The Cer: PE: PC ratio in the plasma membrane is approximately 1:10:30 (82). Moreover, the active site of SMS2 (catalytic triad) is in the outer leaflet of the plasma membrane where PC is enriched and the PE content is very low (85, 86) (Fig. 14). Furthermore, we demonstrated that SMS2 preferentially utilizes PC rather than PE as a substrate in the presence of Cer (Fig. 8). These results strongly suggest that SMS2 acts exclusively as an SMS/PC-PLC but not as a CPES/PE-PLC *in vivo*.

Where does SMS2 exhibit PC-PLC activity rather than SMS activity in the plasma membrane? Surface Cer in the plasma membrane forms large Cer-enriched membrane domains (Cer-rich platforms [CRPs]) that are DRMs (87). At least 5 mol% Cer is required to form Cer-enriched membranes (88). Burgert *et al*. reported that more than 60% of Cer in the plasma membrane is localized to CRPs (89). It has been reported that SMS2 is localized in the membrane microdomains and that approximately 60% of the total SMS2 expressed in the cells was detected in DRM fractions (74), indicating that SMS2 is localized to both CRPs (approximately 60%) (Fig. 14*A*) and membrane regions except CRPs (approximately 40%) (Fig. 14*B*). We demonstrated that the SMS activity of SMS2 surpassed the PC-PLC activity in 1.8 mol% Cer in the presence of 4.7 mol% PC (Fig. 6*C*). These reports and our present results suggest that the major enzyme activity of SMS2 in CRPs is SM synthesis (Fig. 14*A*) and that SMS2 primarily exhibits PC-PLC activity in membrane regions except in CRPs (Fig. 14*B*). Intriguingly, DG inhibited the SMS activity of SMS2 (feedback control) (Fig. 13). Therefore, it is possible that the DG produced by SMS2 itself also regulates the balance between the SMS activity and the PC-PLC activity of SMS2, resulting in PC-PLC being exclusive.

The activity of mammalian PC-PLC and PE-PLC were first reported approximately 70 and 35 years ago, respectively. However, both genes are unidentified (22, 23, 24, 25, 26, 27, 28). PC-PLC activity has been detected in DRMs (47, 48) and PE-PLC activity was detected in membrane fractions in mammalian cells (16, 33, 34, 35, 36). Both PLCs were inhibited by D609 (16, 57, 58, 59, 68, 90, 91) (Table 1). SMS2, which is localized in DRMs in the plasma membrane (74), exhibited D609-sensitive PC-PLC and PE-PLC (Fig. 10*A*), strongly suggesting that SMS2 is a promising candidate for long-sought mammalian PC-PLC and PE-PLC.

Metal ions modulate PLC activities (Table 1). For example, mammalian PIP_2_-PLC is activated by Ca^2+^ (19). Zn^2+^ activates bacterial PC-PLC but inhibits plant PLC (46, 92, 93, 94). Because D609 inhibits bacterial PC-PLC, it has been proposed that D609 acts as Zn^2+^ chelator (44, 58, 59). However, Zn^2+^ strongly inhibited all DG-producing activities of SMS isoforms (Fig. 12 and Table 2) (54, 65). Moreover, PC-PLC activity in bull seminal plasma was inhibited by Zn^2+^ (95). These results and reports imply that the mechanisms of PC hydrolysis by mammalian and bacterial PC-PLCs are distinct from each other and that the mechanisms of action of D609 for SMS2 and bacterial PC-PLC are also different from each other.

Interestingly, Zn^2+^ has an opposite effect on human acid sphingomyelinases (ASMs), which hydrolyze SM to generate Cer (the reverse reaction of SMS) (96, 97). Secretory ASM hydrolyzes SM in the outer leaflets of the plasma membrane. Therefore, Zn^2+^ may act as a key regulator of the balance between Cer and SM in the plasma membrane by oppositely regulating ASM and SMS2 activity. Mg^2+^ slightly inhibited the SMS activity of SMS2 (Fig. 12*B*) but activated the neutral ASM (98), suggesting that Mg^2+^ is also a candidate for regulating the balance between Cer and SM. Moreover, DG was reported to activate ASM activity (99), whereas DG inhibited the SMS activity of SMS2 due to a competitive reaction (Fig. 13*A*), which is PC:DG choline phosphotransferase activity (50). Therefore, the DG also may regulate the balance between Cer and SM in the plasma membrane by regulating ASM and SMS2 activity.

Because we evaluated the enzymological properties of all human SMS families (SMS2 [the present study], SMS1 (54) and SMSr (64, 65)) by absolute quantification of lipid using LC–MS/MS, these properties can be compared quantitatively (Table 2). Intriguingly, the specific activity of SMS2 (lipid production (pmol)/mg protein/min) was approximately 10-fold higher than that of SMS1 (Table 2). Previous studies showed that SMS activity of SMS2 is approximately 2-fold higher than that of SMS1 in mammalian cells (73, 84, 100, 101). Because SMS activity of SMS2 is higher than that of SMS1 (Table 2), it is likely that the higher SMS activity of SMS2 in mammalian cells is due to its intrinsic properties.

The SMSs possess a conserved LPP catalytic triad (20) (Fig. 14 and Fig. S1). This catalytic triad is crucial for the DG-generating activities of SMS1 and SMSr (Table 2) (51, 70, 102). We demonstrated that the catalytic triad (H229, H272, and D276) of human SMS2 is crucial for all DG-generating activities (PC-PLC, PE-PLC, SMS, and CPES activities) (Fig. 9), suggesting that the catalytic triad of SMS2 is a common site for SMS, CPES, PC-PLC, and PE-PLC reactions. Thus, we propose a model of DG-producing reactions, which can be divided into two steps (20) (Fig. 1). In the first step, glycerophospholipids are hydrolyzed, and N–P bonds are established between the free polar head (PCho/PEA) and the histidine of the catalytic triad. In the second step, the N–P bond is cleaved and the O–P bond is formed with the acceptor (water for the PLC reaction and Cer for the SMS/CPES reaction). Because SMS2 displays stronger SMS activity than PC-PLC activity in the presence of PC and Cer (Figs. 3*C* and 4*C*), the accessibility of Cer molecules to the catalytic region of SMS2 may be higher than that of water molecules. However, when Cer concentrations are low, the O–P bond can be formed with water instead of Cer.

It was reported that E271 of SMS2, adjacent to the catalytic histidine (H272) significantly influenced enzyme specificity (CPE production) (53). E271 of SMS2 is crucial for CPES activity (Fig. 9*F*). However, the E271D mutation did not affect other DG-producing activities of SMS2 (Fig. 9, *B* and *D* and G), suggesting that E271 of SMS2 is essential only for CPES reaction in the second step of CPE production (Fig. S1*B*) in addition to the catalytic triad.

We previously demonstrated that purified human SMSr exhibits PAP, PI-PLC, PE-PLC, and PC-PLC activities in addition to weak CPES activity *in vitro* (64, 65) (Table 2). We also reported that SMS1 exhibits PC-PLC, PE-PLC, and CPES activities in addition to SMS activity (54) (Table 2). The enzymatic properties of SMS2, which were revealed in the present study (Figure 3, Figure 4, Figure 5), were more similar to those of SMS1 than to SMSr (Table 2). The amino acid sequence of human SMSr is only approximately 34% identical to that of human SMS1 and SMS2 (50), whereas SMS2 shares 57% amino acid identity with SMS1 (103). These tendencies of sequence dissimilarity and similarity indicate that the enzymatic properties of SMSr are relatively different from those of SMS2 and SMS1, and that those of SMS2 and SMS1 are relatively similar (Table 2).

Several studies on the physiological and pathological functions of SMS2 have been published (55, 103, 104). For example, SMS2 is involved in physiological and pathological processes such as chronic obstructive pulmonary disease (105), atopic dermatitis (106), fetal alcohol spectrum disorder (107), depression (108), atherosclerosis (109, 110), pulmonary edema (111), colitis and colitis-associated cancer (112), middle cerebral artery occlusion (113), type 2 diabetes (insulin resistance and obesity) (74, 78, 114, 115) and liver steatosis (116). Because SMS2 is regarded as SMS (Cer-metabolizing enzyme), many previous studies have primarily focused on sphingolipid metabolism (the balance between Cer and SM) mediated by SMS2 (55, 103, 104). In the present study, we shed new light on SMS2 as PC-PLC, PE-PLC, and CPES. Because the catalytic site of SMS2 (catalytic triad) is located in the outer leaflet of the plasma membrane, where SFA- and/or MUFA-containing PCs are enriched and PE and Cer concentrations are low, SMS2 is likely to act as a PC-PLC in the plasma membrane. Taken together with our previous reports showing that SMS1 and SMSr also act as SFA- and/or MUFA-containing DG-producing enzymes, independent of Cer (54, 65), we propose that all SMS isoforms are categorized as novel mammalian PLCs that comprise SFA- or MUFA-containing DG signaling pathways, independent of PI turnover (2), which is the PUFA (20:4)-containing DG signaling pathway.

## Experimental procedures

### Materials

Antibodies: mouse monoclonal anti-Strep II antibody (M211-3) was obtained from Medical and Biological Laboratories. Peroxidase-conjugated goat anti-mouse IgG was purchased from Bethyl Laboratories (Montgomery). Peroxidase-conjugated goat anti-rabbit IgG antibody (111-036-045) was obtained from Jackson ImmunoResearch (West Grove).

Lipids: 1-palmitoyl-2-oleoyl-*sn* -glycero-3-phosphate (16:0/18:1-PA, cat. no. 840857), 1-palmitoyl-2-oleoyl-*sn*-glycero-3-phosphoinositol (16:0/18:1-PI, Cat. No. 850142), 1-palmitoyl-2-oleoyl-*sn*-glycero-3-phospho-L-serine (16:0/18:1-PS, Cat. no. 840034), and 1-pentadecanoyl-2-oleoyl-*sn*-glycerol (15:0/18:1-DG, Cat. no. 330722), 1-palmitoyl-2-oleoyl-*sn*-glycero-3-PCho (16:0/18:1-PC, Cat. no. 850457); 1-palmitoyl-2-oleoyl-*sn*-glycero-*sn*-glycerol (16:0/18:1-DG, Cat. no. 800815); 1,2-dioleoyl-*sn*-glycero-3-PCho (18:1/18:1-PC; Cat. no. 850375), 1-stearoyl-2-arachidonoyl-*sn*-glycero-3-PCho (18:0/20:4-PC, Cat. no. 850469), 1-palmitoyl-2-oleoyl-*sn*-3-phosphoglycerol (16:0/18:1-PG, Cat. No. 840457), and N-lignoceroyl-D-erythro-sphingosylphosphoethanolamine (d18:1/24:0-CPE, Cat. no. 860067), and N-lauroyl-D-erythro-sphingosylphosphorylcholine (d18:1/12:0-SM, Cat. no. 860583) were purchased from Avanti Polar Lipids (Alabaster). N-stearoyl-D-erythro-sphingosine (d18:1/18:0-Cer, Cat. No. 19556) and N-octadecanoyl sphingosylphosphorylcholine (d18:1/18:0-SM, Cat. no. 24355) were obtained from Cayman Chemical Company.

Detergents: n-dodecyl-β-D-maltoside (DDM) was obtained from Cayman Chemical Company. Cholesteryl hemisuccinate (CHS) was purchased from Sigma-Aldrich.

Compounds: D-Desthiobiotin was purchased from Sigma-Aldrich. Tricyclodecan-9-yl-xanthogenate (D609) was obtained from Chemscene. Linear polyethylenimine hydrochloride (transfection grade) (MW 40,000) was purchased from Polysciences.

### Plasmids

C-terminal TS-tagged Human SMS2 (SGMS2) (NCBI Reference Sequence: NM_001136257.1, UniProt accession number: Q8NHU3) expression plasmid vector was generated (Addgene plasmid # 202529; http://n2t.net/addgene:202,529; RRID:Addgene_202529) (54, 117). Briefly, we used pCAGGS-N-TEV-TS (Addgene plasmid # 202526; http://n2t.net/addgene:202,526; RRID: Addgene_202526), a plasmid expressing the C-terminal TEV protease-cleavable TS-tag (ENLYFQGS-WSHPQFEK-(GGGS)_2_-GGSA-WSHPQFEK) fusion proteins in mammalian cells. The plasmid was linearized using the XhoI restriction enzyme. Full-length SMS2 was amplified using the following primers: forward, 5′-ATCGCGGCCGCTCGAGCACCATGGATATCATAGAG -3’; reverse, 5′- AGAGGTTTTCCTCGAGGGTCGATTTCTCATTGTC -3’. The PCR products were subcloned into pCAGGS-N-TEV-TS by in-fusion cloning (Clontech-Takara Bio).

### Cell culture

HEK293 cells (Japanese Collection of Research Bioresources) were maintained in Dulbecco’s modified Eagle’s medium (Wako Pure Chemicals) supplemented with 5% fetal bovine serum (Thermo Fisher Scientific) and 100 U/ml penicillin/100 μg/ml streptomycin (Wako Pure Chemicals) at 37 °C in an atmosphere containing 5% CO_2_. Plasmids were transiently transfected using PEI Max (#24765-100, Polysciences) (118). Cells (6.25 × 10^5^) were plated on 150-mm dishes. After 96 h, plasmid cDNA (pCAGGS-SMS2-TEV-TS) was transfected with PEI Max. The expression vectors with PEI (1 mg/ml, pH 8.0) were preincubated for 10 min at a 1:3 ratio (15 μg DNA: 45 μl PEI) in 750 μl of Opti-minimal essential medium before transfection. The DNA complex (15 μg DNA) was transfected into HEK293 cells (approximately 50% confluent in a 150 mm dish). After 24 h, the cells were harvested and the pellets were resuspended in 40% (v/v) glycerol diluted in phosphate-buffered saline. The cell samples were flash-frozen in liquid nitrogen and stored at −80 °C until use.

### Purification of TS-tagged proteins

C Terminally TS-tagged human SMS2 (SMS2-TS), SMS2^H229A^-TS, SMS2^H272A^-TS, SMS2^H276A^-TS, and SMS2^E271D^-TS were expressed in HEK 293 cells.

Frozen cells (3.2 × 10^8^ cells) expressing recombinant proteins were thawed at 4 °C. After centrifugation at 10,000×*g* for 30 min at 4 °C, cell pellets were resuspended in ice-cold lysis buffer (20 mM Tris–HCl (pH 7.4), 150 mM NaCl, 1% (w/v) DDM, 0.2% (w/v) CHS, 0.1 mM DTT, 10% (v/v) glycerol, 1 mM PMSF, 20 μg/ml aprotinin, 20 μg/ml leupeptin, and 20 μg/ml pepstatin), and cells were homogenized on ice using a homogenizer (cat. no. 885300-0100, Kimble Kontes) and lysed on ice through sonication. The insoluble material was removed through centrifugation (10,000×*g*, 20 min at 4 °C). The detergent-insoluble pellet was removed by centrifugation (10,000×*g* for 10 min at 4 °C). The supernatant (1% DDM soluble fraction) is isolated by ultracentrifugation (200,000×*g* for 30 min at 4 °C).

The protein was purified using Strep-Tactin XT beads (IBA Life Sciences) (117). The supernatant was loaded onto a column containing 800 μl of Strep-Tactin XT resin. The beads were washed with wash buffer (20 mM Tris–HCl (pH 7.4), 150 mM NaCl, 0.05% (w/v) DDM), 0.01% (w/v) CHS, 0.1 mM DTT, and 10% (v/v) glycerol). TS-tagged proteins were eluted with elution buffer (wash buffer containing 2 mM D-desthiobiotin). Purified samples were concentrated using an Amicon Ultra-15 (Merck).

### Immunoblot analysis

The purified proteins were mixed with a 5 × Laemmli sample buffer containing 2.5% (v/v) 2-mercaptoethanol and incubated at 37 °C for 30 min instead of boiling. The samples were analyzed using SDS-PAGE. TS-tagged proteins were detected by immunoblot analysis as previously described (64, 65, 117). BlueStar Prestained Protein Ladder (#RPN2106, Nippon Genetics) was used as molecular mass markers. Immune complexes were visualized with Amersham enhanced chemiluminescence Western blotting detection reagent (Cytiva) and LuminoGraph I (WSE-6100, ATTO). The proteins were also visualized by Coomassie blue staining (119) and stained gels were scanned using a scanner (GT-X980, Epson). Adjustments to brightness and contrast were applied uniformly across the entire image.

### Preparation of DDM and CHS detergents stock solutions

To prepare the detergent stock solution (10% (w/v) DDM and 2% (w/v) CHS), 5 g of DDM was added to 40 ml of 200 mM Tris–HCl (pH 8.0), followed by gentle rotation until the DDM was added to the solution. The detergent (1 g of CHS) was added to the DDM solution and sonicated until the solution became translucent. After sonication, 200 mM Tris–HCl (pH 8.0) was added up to 50 ml and incubated 25 °C with gently rotation until the solution became transparent. The detergents solution was stored at −25 °C.

### Preparation of micelles and liposomes

The reaction solution for the PLC/SMS/CPES activity assay was prepared as previously described (49, 64, 65, 117). The phospholipids dissolved in chloroform/methanol (2:1 (v/v)) and d18:1/18:0-Cer in chloroform were dried under N_2_ gas to yield a lipid film on the vial wall. The lipid film was resuspended in the reaction buffer (20 mM Tris–HCl (pH 7.4), 150 mM NaCl, 0.05% (w/v) DDM, 0.01% (w/v) CHS, 100 μM DTT, and 10% (v/v) glycerol) to a final total lipid concentration of 100 μM each. When the experiments were conducted without detergent, the lipids were resuspended in reaction buffer without detergent. The reaction solutions containing lipids were vortexed for 3 min and sonicated in a bath sonicator (Sonifier Model 450, Branson) four times for 3 min each at 65 °C. Liposomes with an average diameter of 400 nm were obtained using a Mini Extruder (Avanti Polar Lipids).

### *In vitro* PLC/SMS/CPES assay

DG-generating activities (PLC, SMS, and CPES) of the purified proteins were evaluated using previously reported methods (65, 120, 121). The purified SMS2 samples (10 μl containing approximately 500 ng of purified proteins) were diluted in 10 μl ice-cold reaction buffer on ice (final concentration of 20 mM Tris–HCl (pH 7.4), 150 mM NaCl, 0.05% (w/v) DDM, 0.01% (w/v) CHS, 0.1 mM DTT, 10% (v/v) glycerol, and 50 μM phospholipids (16:0/18:1-PC, 16:0/18:1-PE, 16:0/18:1-PA, 16:0/18:1-PI, 16:0/18:1-PS, or 16:0/18:1-PG). For the SMS/CPES activity assay, d18:1/18:0-Cer was added to the reaction buffer at final concentration of 50 μM. As SMS2 is a membrane-associated and lipid-dependent enzyme, a surface dilution kinetic scheme was used (122, 123). The mol % of lipids in the DDM/CHS/lipid mixed micelles was calculated using the formula: mol% = 100 × [lipid (mol)]/([DDM (mol)] + [CHS (mol)] + [lipid (mol)]) (122, 123). In this assay, the surface concentration of phospholipids (50 μM) in the micelles (0.81 mM DDM and 0.21 mM CHS mixed micelle) is 4.70 mol%. After incubation (enzyme reaction) for 2 h at 37 °C, the assay samples were stored at −80 °C until the measurements.

### Lipid quantification using LC–MS/MS

Lipids in the samples were extracted using the Bligh and Dyer method (49, 64, 65, 124). Briefly, samples (20 μl) were added to 380 μl of water. To compensate for any possible variation during the entire process (*e*.*g*.*,* recovery rate of lipids in lipid extraction step, HPLC injection, and ionization variability) (69), I.S.: 20 ng each of 15:0/18:1-DG, d18:1/12:0-SM, and d18:1/24:0-CPE were added from this step. The samples were mixed with methanol (1 ml) and chloroform (0.5 ml). After vortexing for 30 s, samples were incubated for 5 min at room temperature (25 °C). The samples were then mixed with chloroform (0.5 ml) and water (0.5 ml) and vortexed for 30 s. After centrifugation (1000×*g*, 25 °C, 10 min), the lower phase containing extracted lipids was transferred to a new test tube and used for quantitation of lipids (DG, SM, and CPE) *via* LC‒MS/MS (49, 64, 65).

Ionized DG ([M + NH4]^+^), SM ([M + H]^+^), and CPE ([M + H]^+^) were isolated in the first quadrupole (Q1). Thereafter, the product ions of the DG species [33], SM species (*m/z* 184.1 in positive ion mode), or CPE species ([M + H −141.0]^+^) were reselected at Q3 after fragmentation at Q2 by collision-induced dissociation.

The peak regions corresponding to analyte signals from chromatograms were selected, followed by the integration of peak areas for each lipid multiple reaction monitoring transition to calculate the peak area of analytes using the MultiQuant software (AB SCIEX). The amounts of detected lipids were normalized by calculating peak area ratio of the analyte to the I.S. (Peak Area Ratio), enabling relative quantification (69).

For absolute quantification of lipids, we generated calibration curves of Peak Area Ratio using commercially available purified lipids (16:0/18:1-DG and d18:1/18:0-SM) (Fig. S2). Using calibration curves, we determined the absolute amounts of 16:0/18:1-DG and d18:1/18:0-SM in samples (mole/sample). Unfortunately, purified d18:1/18:0-CPE is unavailable. Thus, we compared CPE-producing activity using peak area ratio.

To distinguish between the PC-PLC activity (product: DG) and SMS activity (products: SM and DG) of SMS2 in the presence of 16:0/18:1-PC and d18:1/18:0-Cer (Fig. 1), the subtracted values (16:0/18:1-DG – d18:1/18:0-SM) were used for PC-PLC activity. To distinguish between the PE-PLC activity (product: DG) and CPES activity (products: CPE and DG) of SMS2 in the presence of 16:0/18:1-PE and d18:1/18:0-Cer (Fig. 2), the subtracted values (16:0/18:1-DG – d18:1/18:0-CPE) were used for PC-PLC activity. The produced DG, SM, and CPE by purified SMS2 were calculated after subtracting the background samples (purified SMS2 and phospholipids without incubation).

### SMS2 reconstitution into liposomes

To generate proteoliposomes (125), purified SMS2 was reconstituted into liposomes. 18:1/18:1-PC dissolved in chloroform/methanol (2:1 (v/v)) was dried under N_2_ gas to yield a lipid film on the vial wall. The lipids were resuspended in a buffer containing.

Tris–HCl (20 mM, pH 7.4), 150 mM NaCl, 100 μM DTT, 10% (v/v) glycerol (final concentration of 18:1/18:1-PC is 0.255 mM). The reaction solutions were vortexed for 3 min and sonicated in a bath sonicator for 3 min each at 65 °C. SMS2-TS was purified from HEK293 cells. To remove detergent *via* dialysis, 1 ml of elution buffer containing 0.01% DDM and 0.002% CHS was used for elution from the resin. The SMS2-TS containing sample (50 μg proteins/ml, 0.5 ml) was mixed with liposome (1 ml). The solution was diluted with 6 ml buffer without liposomes to reduce the detergent content below the critical micelle concentration of DDM is approximately 0.0087%. The lipid-to-protein molar ratio was set to 500:1 (200 μg lipid to 21.4 μg protein). Detergents were removed by dialysis against 1 L of 20 mM Tris–HCl (pH 7.4), 150 mM NaCl, 100 μM DTT, and 10% (v/v) glycerol for 31 h at 4 °C with five buffer changes. To evaluate PLC/SMS/CPES activity in the absence of detergents, 50 μM liposome (18:1/18:1-PC: 16:0/18:1-glycerophospholipid and/or d18:1/18:0-Cer = 9:1) was used as substrate.

### Cell-free expression of SMS2

The C-terminal TS-tagged human SMS2 was subcloned into the pT7-IRES vector using in-fusion cloning (Clontech, Takara Bio) and BamHI. The plasmid and human cell-free protein expression system (cat. no. 3281, Clontech, Takara Bio) and SMS2-TS was expressed *in vitro* according to the manufacturer’s instructions. SMS2-TS is expressed in the presence of 18:1/18:1-PC liposomes (11.6 μM). The mixture containing cell lysate (80 μl) was mixed with 8 μl of liposome (1 mg/ml 18:1/18:1-PC). Translation reactions were performed for the PLC/SMS/CPES enzyme activity assay without detergents. The expression of SMS2-TS was determined by immunoblotting using a mouse monoclonal anti-Strep II tag antibody. Since the SMS2-TS–expressing sample mixture was used without protein purification, the mixture of the cell-free expression system with the pT7-IRES vector alone was used as a negative control.

### Statistical analysis

Data are presented as means ± SD and were analyzed using Student’s *t* test (two-tailed, unpaired) for the comparison of two groups or one-way ANOVA with followed by Tukey’s post hoc test (for comparing every mean with every other mean) or Dunnett’s post hoc test (for comparing compare every mean to a control mean) using GraphPad Prism 9 (GraphPad Software) to determine any significant differences. Individual data points (technical replicates) are superimposed on all the bar graphs. Statistical significance was set at *p* < 0.05.

## Data availability

Data supporting the findings of this study are available from the corresponding author upon request.

## Supporting information

This article contains supporting information (20, 21, 126, 127).

## Conflict of interest

The authors declare that they have no conflicts of interest with the contents of this article.
