# Supplementary material for: Multiple activities of sphingomyelin synthase 2 generate saturated fatty acid– and/or monounsaturated fatty acid–containing diacylglycerol
Source: J Biol Chem. 2024 Nov 5;300(12):107960. doi: 10.1016/j.jbc.2024.107960 (PMC11663969; doi:10.1016/j.jbc.2024.107960)
Supplement: Supplemental Figure Legends [file mmc1.docx]

**Figure S1. Proposed catalytic mechanism for PLC, SMS, and CPES reactions of human SMS2**

DG generation by SMS2 proceeds in two steps, which is similar to the phosphatase reaction of LPP (20, 21). (A) The first step involves a charge relay system (127, 128). This step serves to conduct electrons through hydrogen bonds from Asp-276 through His-272, thereby forming an N–P bond between His-272 and the polar head of phospholipids. The catalytic histidine (His-229) facilitates the cleavage of the phosphodiester bond of phospholipids and release of DG by acting as a general acid. (B) In the second step, His-229 acts as a general base to facilitate the cleavage of the N–P bond. The water molecule (PLC reaction) or ceramide (SMS or CPE reaction) serve as general acids.

**Figure S2. Calibration curves of 16:0/18:1-DG and d18:1/18:0-SM by using LC-MS/MS**

The calibration curves of 16:0/18:1-DG (A) and d18:1/18:0-SM (B) were generated using commercially available purified lipids (see Materials). Lipids were dissolved in 500 µl of chloroform/methanol (2:1, v/v) and 200 µL of samples were transferred to a vial. The internal standards (40 ng each of 15:0/18:1-DG and d18:0/12:0-SM) were added (final concentration is 0.2 ng/µL) and 10 µL were analyzed by LC-MS/MS. Ionized DG species ([M + NH4]+), SM species ([M + H]+) were isolated at the first quadrupole (Q1). Thereafter, a product ion of DG species (m/z 339.3 in positive ion mode), SM species (m/z 184.1 in positive ion mode)

The standard curves were established using different amounts of samples (0–1000 pg/µL). 16:0/18:1-DG: y= 0.004105x + 0.0084, R^2^=0.9995; d18:1/18:0-SM: y= 0.002705x – 0.00147, R^2^=0.994.
