# Supplementary material for: Multiple activities of sphingomyelin synthase 2 generate saturated fatty acid– and/or monounsaturated fatty acid–containing diacylglycerol
Source: J Biol Chem. 2024 Nov 5;300(12):107960. doi: 10.1016/j.jbc.2024.107960 (PMC11663969; doi:10.1016/j.jbc.2024.107960)
Supplement: Supplemental Figures [file mmc2.pdf]

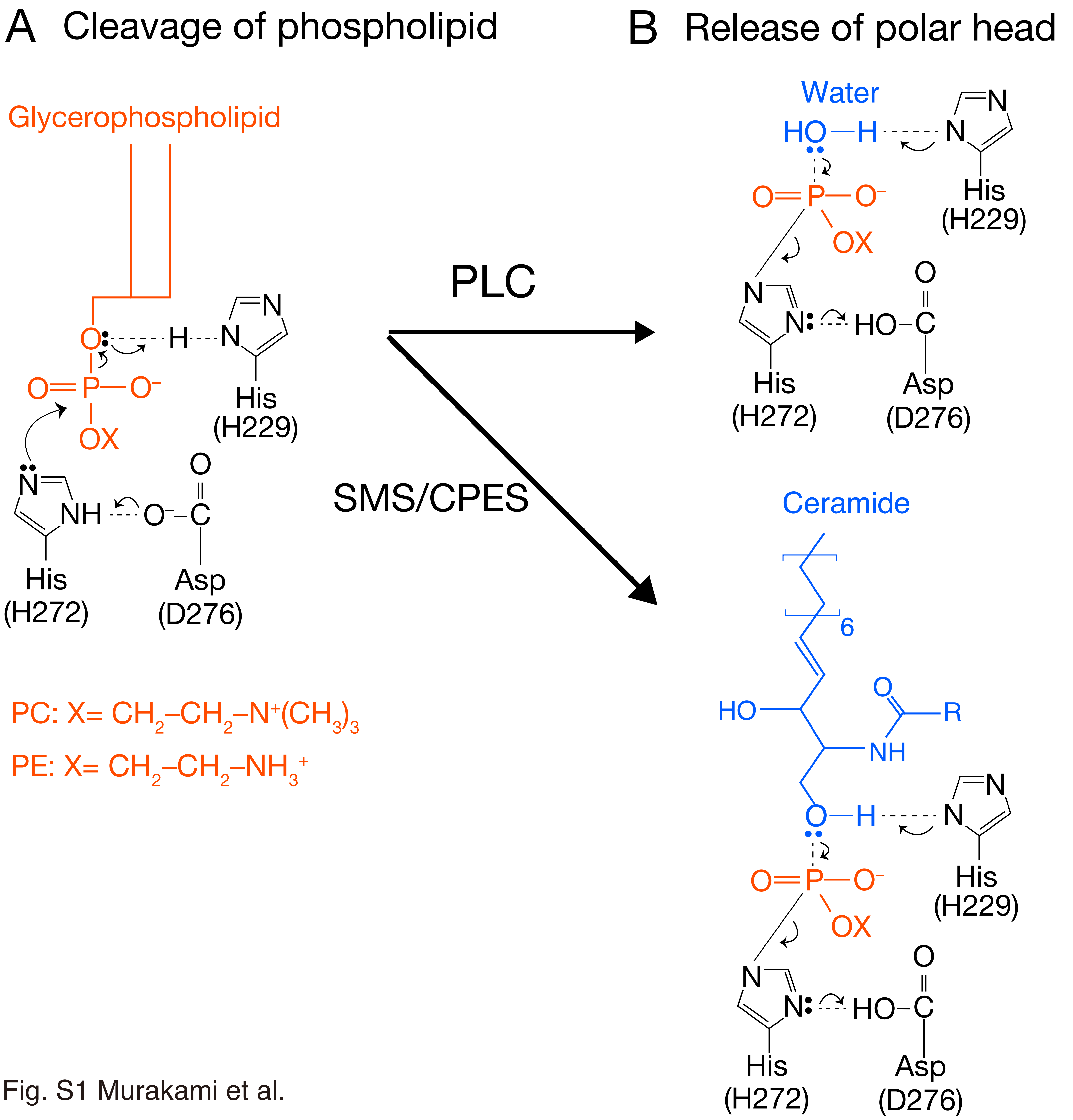

Fig. S1 Murakami et al.

A

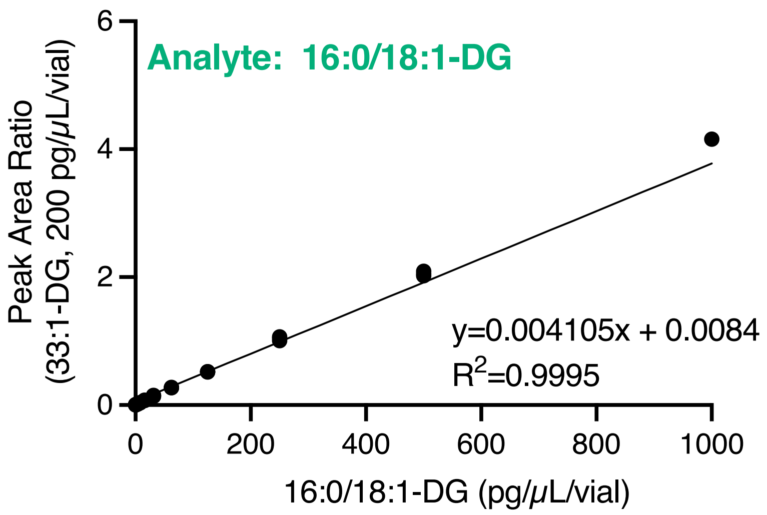

B

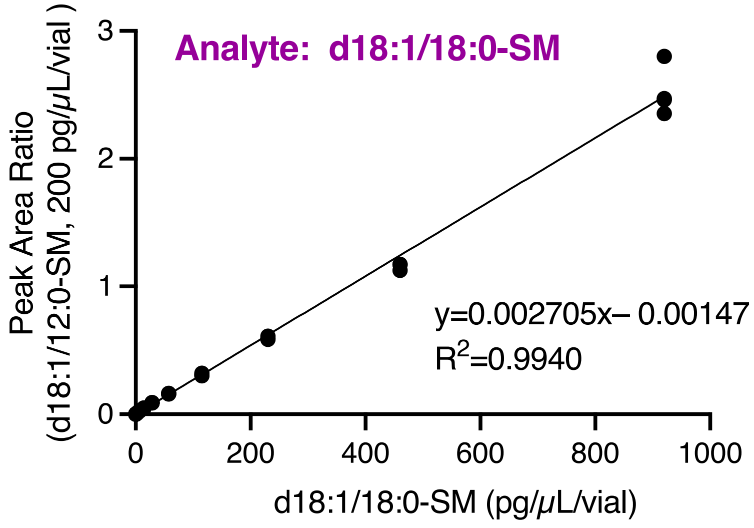

Fig. S2 Murakami et al.
